# Chelator impact: investigating the pharmacokinetic behavior of copper-64 labeled PD-L1 radioligands

**DOI:** 10.1186/s41181-024-00243-5

**Published:** 2024-02-19

**Authors:** Fabian Krutzek, Cornelius K. Donat, Sven Stadlbauer

**Affiliations:** 1 Institute of Radiopharmaceutical Cancer Research, Helmholtz-Zentrum Dresden-Rossendorf, Bautzner Landstrasse 400, 01328 Dresden, Germany; 2 School of Science, Faculty of Chemistry and Food Chemistry, Technical University Dresden, 01069 Dresden, Germany

**Keywords:** PET-tracers, Small molecules, Copper-64, Chelators, PD-L1

## Abstract

**Background:**

Programmed cell death ligand 1 (PD-L1) plays a critical role in the tumor microenvironment and overexpression in several solid cancers has been reported. This was associated with a downregulation of the local immune response, specifically of T-cells. Immune checkpoint inhibitors showed a potential to break this localized immune paralysis, but only 30% of patients are considered responders. New diagnostic approaches are therefore needed to determine patient eligibility. Small molecule radiotracers targeting PD-L1, may serve as such diagnostic tools, addressing the heterogeneous PD-L1 expression between and within tumor lesions, thus aiding in therapy decisions.

**Results:**

Four biphenyl-based small-molecule PD-L1 ligands were synthesized using a convergent synthetic route with a linear sequence of up to eleven steps. As a chelator NODA-GA, CB-TE2A or DiAmSar was used to allow radiolabeling with copper-64 ([^64^Cu]Cu-**14**–[^64^Cu]Cu-**16**). In addition, a dimeric structure based on DiAmSar was synthesized ([^64^Cu]Cu-**17**). All four radioligands exhibited high proteolytic stability (> 95%) up to 48 h post-radiolabeling. Saturation binding yielded moderate affinities toward PD-L1, ranging from 100 to 265 nM. Real-time radioligand binding provided more promising *K*_D_ values around 20 nM for [^64^Cu]Cu-**14** and [^64^Cu]Cu-**15**. In vivo PET imaging in mice bearing both PC3 PD-L1 overexpressing and PD-L1-mock tumors was performed at 0–2, 4–5 and 24–25 h post injection (p.i.). This revealed considerably different pharmacokinetic profiles, depending on the substituted chelator. [^64^Cu]Cu-**14**, substituted with NODA-GA, showed renal clearance with low liver uptake, whereas substitution with the cross-bridged cyclam chelator CB-TE2A resulted in a primarily hepatobiliary clearance. Notably, the monomeric DiAmSar radioligand [^64^Cu]Cu-**16** demonstrated a higher liver uptake than [^64^Cu]Cu-**15**, but was still renally cleared as evidenced by the lack of uptake in gall bladder and intestines. The dimeric structure [^64^Cu]Cu-**17** showed extensive accumulation and trapping in the liver but was also cleared via the renal pathway. Of all tracer candidates and across all timepoints, [^64^Cu]Cu-**17** showed the highest accumulation at 24 h p.i. in the PD-L1-overexpressing tumor of all timepoints and all radiotracers, indicating drastically increased circulation time upon dimerization of two PD-L1 binding motifs.

**Conclusions:**

This study shows that chelator choice significantly influences the pharmacokinetic profile of biphenyl-based small molecule PD-L1 radioligands. The NODA-GA-conjugated radioligand [^64^Cu]Cu-**14** exhibited favorable renal clearance; however, the limited uptake in tumors suggests the need for structural modifications to the binding motif for future PD-L1 radiotracers.

**Supplementary Information:**

The online version contains supplementary material available at 10.1186/s41181-024-00243-5.

## Background

The tumor microenvironment (TME) operates as a dynamic and immunological network, involving a variety of actors, such as T- and B-cells, natural killer cells (NK cells), and endothelial cells, which interact with both the extracellular matrix and cancer cells (Balkwill et al. 2012; Anderson and Simon 2020). Moreover, the tumor microenvironment often presents biological structures that can be targeted using molecular imaging techniques. Potentially, this allows an early detection of tumors, or a staging, thus aiding treatment decisions and allowing the monitoring of therapeutic strategies. The programmed cell death ligand 1, PD-L1 (CD274) is an example of a target that has gained considerable attention in recent years. Its interaction with PD-1 (CD279) results in an immune checkpoint, regulating the immune response under homeostatic conditions, thereby preventing autoimmunity effects (Santini and Hellmann 2018; Han et al. 2020). Malignant tumors exploit these regulatory points by overexpressing the associated targets, such as PD-L1, thereby evading the local immune response in the TME. Blocking PD-1 or PD-L1 with inhibitors represents a promising therapeutic strategy, as reactivation of the local immune system in the TME leads to the identification and targeting of malignant tissue, e.g. via T-cells (Budimir et al. 2022). Antibodies constitute the dominant structural class for this type of treatment. However, antibody production and therapies cause substantial medical care costs and were found to be associated with several adverse effects, for instance caused by immunogenicity (Wu et al. 2022). Most importantly, PD-L1 antibody therapies resulted in low response rates of about 30% (Farid 2007; Guardascione and Toffoli 2020; Huang et al. 2020). Small-molecule inhibitors could address some of these problems, being cost-effective and less likely to cause immunogenic adverse effects. Currently, there are only two small molecule-based inhibitors in phase 1 clinical trials, INCB086550 (Koblish et al. 2022) and GS-4224 (GileadSciences 2022). One reason for the low response rates is the heterogeneous expression of PD-L1 between and within tumor lesions, leading to potentially inaccurate PD-L1 detection results (Yi et al. 2018). At present, patient classification and treatment decisions rely on biopsies and immunohistochemical staining, which can cause distress for patients when performed regularly (Munari et al. 2017). Noninvasive molecular imaging approaches, such as positron emission tomography (PET) and single-photon emission computed tomography (SPECT), offer the possibility to accurately and quantifiably assess PD-L1 expression over time (Rudin and Weissleder 2003). This can eventually support patient stratification and thus increase success rates of checkpoint inhibitor therapies.

To date, a variety of radiotracers targeting PD-L1 or PD-1 have been developed (Krutzek et al. 2022). These include large and midsized constructs, such as antibodies (Heskamp et al. 2015; Kikuchi et al. 2017; Li et al. 2018; Jagoda et al. 2019), nanobodies (Broos et al. 2017; Bridoux et al. 2020), and adnectines (Donnelly et al. 2018; Stutvoet et al. 2020). Additionally, smaller structures like peptides (Kuan et al. 2019; Zhang et al. 2023) and small molecules (Miao et al. 2020; Maier et al. 2022; Xu et al. 2023) were described. Antibody-based radioligands have demonstrated promising results, mainly through their high affinity and were already employed in patients. However, they exhibit long circulation times (Pollack et al. 2018), necessitating the use of long-lived radioisotopes and thereby increasing the patient's radiation burden.

In contrast, peptides and small molecules show substantial promise as imaging agents due to their enhanced tissue and tumor penetration and rapid clearance (Adams et al. 2015). This combination can result in remarkable imaging contrast within a short time. The cyclic peptide WL12 is such an example, showing excellent affinity and specificity, along with suitable pharmacokinetics in vivo (Chatterjee et al. 2017; De Silva et al. 2018; Lesniak et al. 2019). Hence, this peptide has recently been advanced to clinical trials, marking a significant step forward in this field (Zhou et al. 2022). In contrast, small molecule-based PD-L1 radiotracers yielded fewer promising outcomes (Ważyńska et al. 2023), mainly caused by high nonspecific binding, relatively low in vivo tumor uptake, and primarily hepatobiliary clearance (Miao et al. 2020; Maier et al. 2022; Xu et al. 2023). Some of these problems could potentially be ameliorated via different radiolabeling strategies. While covalently binding radionuclides like ^11^C or ^18^F are usually an excellent choice for small molecules, this labeling approach also has some drawbacks, e.g. low tolerance of functional groups with acidic protons (Halder and Ritter 2021; Ajenjo et al. 2021). The clinically applied PSMA and FAPI small molecule radioligands have shown that a chelator-based labeling with radiometals is a suitable alternative (Gourni and Henriksen 2017; Lindner et al. 2021). In this regard, chelators can be used to modify the ligands’ pharmacokinetic profile. They can introduce a notable hydrophilicity to the molecule, potentially leading to faster clearance, ideally via the renal pathway. However, the employed radiometal does influence the choice of available chelators. The cyclotron-produced radionuclide ^64^Cu stands out due to its low positron emission energy of *E*_β+_  = 0.65 MeV and the absence of abundant gamma emission, which results in excellent image quality (Sun and Anderson 2004; Jalilian and Osso 2017). The half-life of *t*_1/2_ = 12.7 h hours provides convenient handling and a wide selection of both commercially and noncommercially chelators (Uzal-Varela et al. 2022).

These chelators are based on various scaffolds, such as 1,4,7-triazacyclononanes (e.g. NOTA (Price and Orvig 2014)), cyclams (e.g. CB-TE2A (Wadas and Anderson 2006)), or sarcophagins [e.g. DiAmSar (Uzal-Varela et al. 2022; Bottomley et al. 1994; Anderson and Ferdani 2009)]. When selecting a chelator, considerations should encompass not only thermodynamic and kinetic stability but also factors like hydrophilicity and the charge of the chelator. These aspects play substantial roles in the process of radiotracer development (Raheem et al. 2023).

In this study, we report on the design and convergent synthesis of four PD-L1 ligands, which were subsequently radiolabeled with ^64^Cu. We tested their in vitro stability and explored the influence of the chelator on the in vitro and in vivo pharmacokinetic profile by modifying the charge of the radioligand and its hydrophilicity. These findings offer valuable insights for future radioligand development endeavors, particularly for the development of small molecule-based PD-L1 radioligands.

## Results

### Design of PD-L1 radioligands

In our previous work (Krutzek et al. 2023a, b, c), we reported on dimethyl biphenyl based PD-L1 ligands exhibiting high binding affinities toward PD-L1. For achieving the desired renal clearance, incorporation of three sulfonic acids proved to be most advantageous (Krutzek et al. 2023b). However, as sulfonic acids lead to increased circulation times of the tracer due to albumin binding, we partially replaced sulfonic acids with phosphonic acids. But that resulted in undesired accumulation in liver, bones, bone marrow and joints (Krutzek et al. 2023c). Therefore, for the present study we chose PD-L1 ligands bearing three sulfonic acids in order to investigate the effect of the chelator on pharmacokinetic behavior. Our rationale was that, depending on the type of chelator, lipophilicity could be mitigated, thus reducing nonspecific binding/uptake, e.g. in the liver and improve renal clearance.

Three different chelators NODA-GA, CB-TE2A, and DiAmSar – with excellent kinetic stabilities were utilized to investigate their impact on the pharmacokinetic behavior of these PD-L1 radioligands. Upon complexation with ^64^Cu, these chelators show different charges: NODA-GA features three carboxylic acid groups, which result in an overall negative net charge of the radioligand. CB-TE2A, a cross-bridged cyclam-based chelator possesses only one free carboxylic acid group, which results in a net charge of + 1 for the radioligand. In contrast, the DiAmSar chelator confers a net charge of + 2 (for dimeric conjugation) or + 3 (for monomeric conjugation) to the binding motif. For CB-TE2A and DiAmSar chelators, the positive charge(s) serve to counterbalance the negative charges from the sulfonic acids present in the binding motif.

### Synthesis

**Scheme 1:**
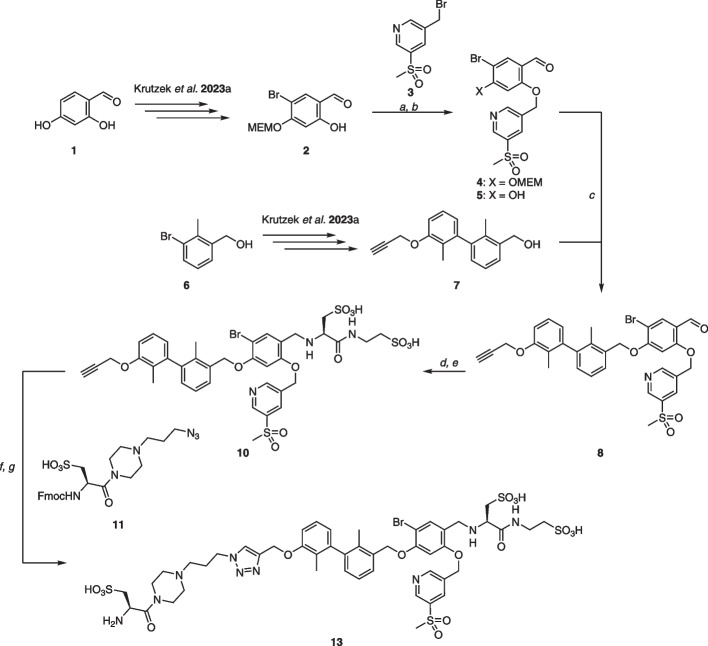
Synthesis of key amine intermediate **13**. **a 3**, K_2_CO_3_, abs. DMF, r.t., 16 h, 81%; **b**, abs. DMF/MeOH (1:1), r.t., 16 h, 41%; **c** DMEAD, PPh_3_, abs. DMF/THF (1:1), 0 °C to r.t., 16 h, 65%; **d** l-cysteic acid, NaBH_3_CN, abs. DMF/MeOH (1:1), r.t., 16 h, 41%; **e** taurine, HATU, HOBt, DIPEA, abs. DMF, r.t., 2 h, 70%; **f 11**, sodium ascorbate, CuSO_4_, THPTA, ^*t*^BuOH/H_2_O (1:1), r.t., 16 h, 78%; **g** NaN_3_, abs. DMF, 50 °C, 3 h, 60%

The synthesis of chelator-bearing PD-L1 ligands followed our previously established convergent synthetic pathway (Krutzek et al. 2023a) (see Scheme 1). The synthesis started with bromination of phenol **2** at the 5-position, followed by selective MEM-protection of the hydroxy group at the 4-position. Nucleophilic substitution involving bromide **3** and subsequent TFA-mediated MEM cleavage produced phenol **5** in a yield of 77% over two steps. The formation of the benzylic ether bond was accomplished with a Mitsunobu reaction of biaryl **7**, derived from bromide **6**. Alkyne **10** was obtained via reductive amination with l-cysteic acid, followed by amide bond formation with taurine. This intermediate underwent a copper-catalyzed azide-alkyne cycloaddition (CuAAC) with azide **11**, yielding **12** in 78% yield. Cleavage of the Fmoc protecting group was achieved with sodium azide at 50 °C in DMF, resulting in the generation of key intermediate **13** for subsequent chelator conjugation.

For accessing the NODA-GA-bearing PD-L1 ligand, **13** was reacted with (^t^Bu_3_)-NODA-GA (HBTU/HOBt, DIPEA), purified and then subjected to the TFA-mediated removal of *tert*-butyl groups, yielding **14** in a moderate yield of 20% over two steps (see Scheme 2). The conjugation with commercially available cross-bridged cyclam derivate CB-TE2A was performed with a good yield of 84%. For the DiAmSar-carrying ligand **16**, the amine **13** was reacted with glutaric anhydride to allow conjugation with the primary amine of the sarcophagin-chelator. Upon conjugation, **16** was isolated in 77% yield, along with a small quantity (9%) of double-conjugated derivative **17**. NMR- and MS-Spectra as well as HPLC chromatograms are reported in the Additional file 1: Supplementary Material as figures S1-S26.

**Scheme 2:**
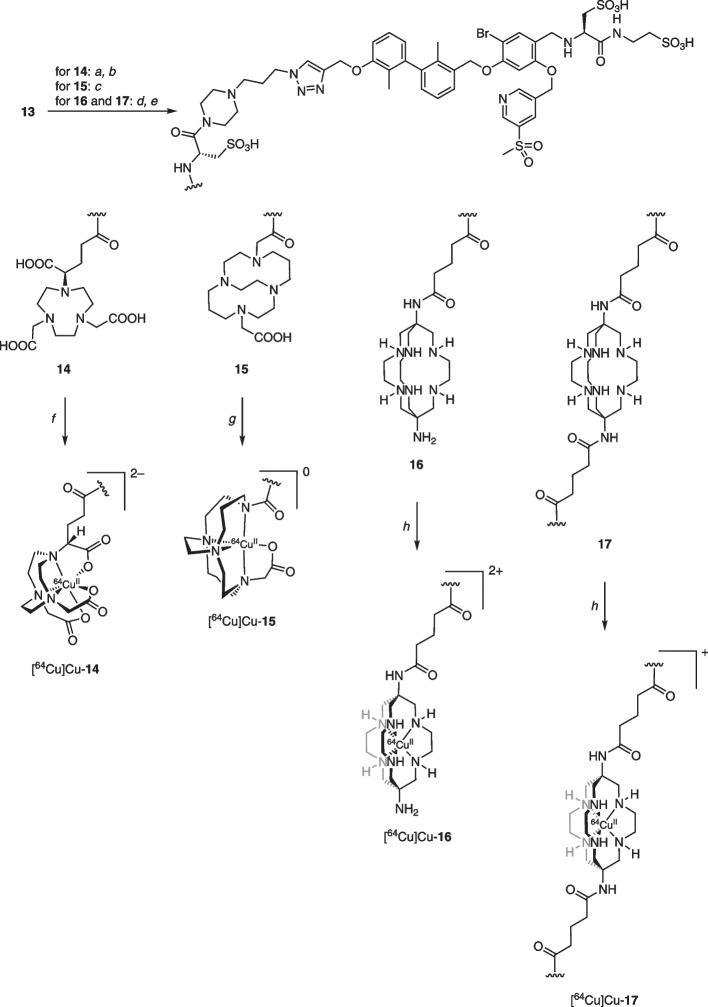
Conjugation of **13** with its respective chelator and their labelling with ^64^Cu. **a** (^*t*^Bu_3_)-NODA-GA, HATU, HOBt, DIPEA, abs. DMF, r.t., 3 h, 46%; **b 14a**, TFA/CH_2_Cl_2_/TES/H_2_O (20:20:8:7), r.t., 40 h, 44%; **c** CB-TE2A, HATU, HOBt, DIPEA, abs. DMF, r.t., 5 h, 85%; **d** glutaric anhydride, DIPEA, abs. DMF, r.t., 16 h, 84%; **e 16a**, DiAmSar, HATU, HOBt, DIPEA, abs. DMF, r.t., 3 h, 77% for **16** and 9% for **17**; **f** [^64^Cu]CuCl_2_, 1 M HEPES (adjusted to pH 4.5 with 1 M HCl), 50 °C, 10 min; **g** [^64^Cu]CuCl_2_, 1 M HEPES (adjusted to pH 4.5 with 1 M HCl), 95 °C, 60 min; **h** 1 M HEPES, r.t., 10 min

### Radiochemistry

**Fig. 1:**
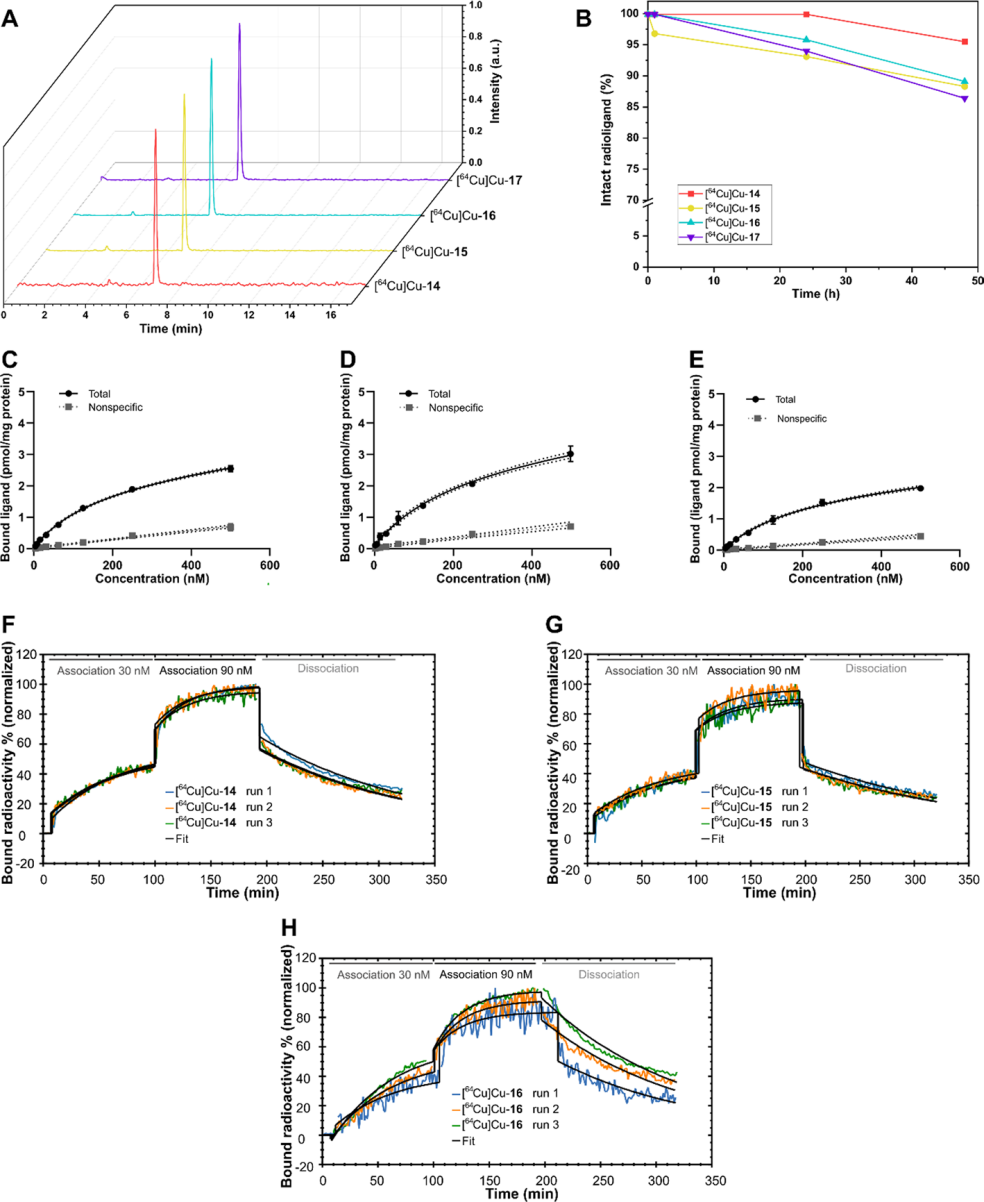
**A** Radio-HPLC chromatograms of radioligands [^64^Cu]Cu-**14**–[^64^Cu]Cu-**17**; **B** Percentage of intact radioligand [^64^Cu]Cu-**14**–[^64^Cu]Cu-**17** over time after incubation in human serum; **C**–**E** Non-linear iterative curve fitting of saturation binding data for radioligands [^64^Cu]Cu-**14**–[^64^Cu]Cu-**16** with dotted lines representing 95% confidence intervals; **F**–**H** Real-time radioligand binding (trace) of compounds [^64^Cu]Cu-**14**–[^64^Cu]Cu-**16**

Radiolabeling of NODA-GA (**14**), CB-TE2A (**15**) and DiAmSar (**16** and **17**) derivatives was performed by modifying previously reported techniques (Sprague et al. 2004; Cai et al. 2009). The NODA-GA- and CB-TE2A-derivative were labeled in 1 M HEPES solution (adjusted to pH 4.5) at 50 °C for 15 min and 95 °C for 60 min, respectively. The DiAmSar-conjugates showed complete chelation of ^64^Cu after incubation in 1 M HEPES solution at room temperature for 15 min. To assess labeling efficiencies, radio-HPLC (Fig. 1A) was conducted, revealing the presence of the radioligands [^64^Cu]Cu-**14**–[^64^Cu]Cu-**17** with retention times of 6.98, 7.15, 6.96 and 7.06 min, respectively. The molar activities used for in vitro and in vivo experiments of the radioligands [^64^Cu]Cu-**14**—[^64^Cu]Cu-**17** were between A_M_ = 12—15 GBq/µmol.

Proteolytic stability studies were performed by incubating the corresponding radioligand in human serum at 37 °C and probing stability via radio-HPLC over a 48-h time course (Fig. 1B). At timepoints 1, 24, and 48 h, an aliquot was withdrawn, proteins precipitated, and the resulting supernatant injected into the HPLC system. The NODA-GA-conjugated radioligand [^64^Cu]Cu-**14** exhibited the highest proteolytic stability, with over 95% of the compound remaining intact after 48 h. The other radioligands—specifically, the CB-TE2A-derivative [^64^Cu]Cu-**15** and both DiAmSar-conjugates—demonstrated similar kinetic stabilities. After 48 h of incubation, these ligands retained 88%, 89%, and 86% of their integrity, respectively.

The partition coefficients (log *D*_7.4_) between *n*-octanol and PBS-buffer pH 7.4 were assessed for all ^64^Cu-labeled compounds using the shake flask technique (Table 1). The resulting log *D*_7.4_ values were –3.00 for the NODA-GA-derivative [^64^Cu]Cu-**14**, –2.11 for CB-TE2A-conjugated [^64^Cu]Cu-**15** and –2.79 for monomeric DiAmSar-complex [^64^Cu]Cu-**16**. For the dimeric DiAmSar ligand [^64^Cu]Cu-**17** determination of Log *D*_7.4_ was unsuccessful due to significant plastic binding of the radioligand even to protein LoBind® Eppendorf tubes.

**Table 1:** In vitro data (^1^saturation binding assay, ^2^real-time radioligand binding) for the ^64^Cu-labeled compounds [^64^Cu]Cu-**14**–[^64^Cu]Cu-**16** and their respective Log *D*_7.4_ values

Compound	*K*_D_ [nM]^1^	B_max_ [pmol/mg]	*K*_D_ [nM]^2^	*k*_a_ (M × s)^–1^	*k*_d_ s^–1^	Log *D*_7.4_
[^64^Cu]Cu-**14**	100.4 ± 9.23	2.40 ± 0.48	23.3 ± 1.67	(5.06 ± 0.25) × 10^3^	(1.17 ± 0.06) × 10^–4^	–3.00 ± 0.03
[^64^Cu]Cu-**15**	184.8 ± 15.4	2.58 ± 0.35	19.1 ± 1.69	(5.10 ± 0.55) × 10^3^	(0.96 ± 0.02) × 10^–4^	–2.11 ± 0.02
[^64^Cu]Cu-**16**	265.0 ± 12.5	3.30 ± 0.46	24.2 ± 0.93	(5.55 ± 0.31) × 10^3^	(1.35 ± 0.09) × 10^–4^	–2.79 ± 0.02

### In-vitro studies

The binding affinities were assessed using two distinct methods. As previously described (Krutzek et al. 2023a, b, c), these biphenyl-based ligands exhibit notable albumin binding properties. However, addition of 2.5% *w*/*v* bovine serum albumin to the assay buffer was necessary to prevent adherence to the plastic of the well plates. That resulted in a compounded affinity measurement of both the specific (PD-L1) and nonspecific (albumin) target. To ascertain binding affinities exclusively toward PD-L1, a real-time ligand binding assay (LigandTracer®) was employed.

From the saturation binding (Fig. 1C–E, Table 1), [^64^Cu]Cu-**14** exhibited the highest affinity (*K*_D_ = 100 nM), followed by the CB-TE2A-derivative [^64^Cu]Cu-**15** (*K*_D_ = 185 nM) and the monomeric DiAmSar-conjugate [^64^Cu]Cu-**16** (*K*_D_ = 265 nM). The B_max_ values were quite similar among all radioligands, ranging from 2.40 pmol/mg protein for [^64^Cu]Cu-**14** to 3.30 pmol/mg protein for [^64^Cu]Cu-**16**. A strong adherence of the radioligand to plastic, specifically for compounds [^64^Cu]Cu-**16** and [^64^Cu]Cu-**17** complicated in vitro measurements. For [^64^Cu]Cu-**16**, this is exemplified by the high residual nonspecific binding (Fig. 1E) in the saturation binding assay. In case of [^64^Cu]Cu-**17**, very high nonspecific binding prevented determination of binding affinity.

Using real-time radioligand binding without BSA (Fig. 1F–G, Table 1), binding affinities were found to be higher for the derivatives [^64^Cu]Cu-**14**–[^64^Cu]Cu-**16**, with *K*_D_ values of 23.3, 19.1 and 24.2 nM, respectively. Coating of petri dishes with collagen was required for reliable real-time binding of [^64^Cu]Cu-**16**. However, despite these conditions no *K*_D_ value was obtained for the DiAmSar-conjugate [^64^Cu]Cu-**17**, due to heavy plastic binding at the cell-free part of the petri-dish.

Additionally, we investigated cell uptake and specificity of binding over time for compounds [^64^Cu]Cu-**14** and [^64^Cu]Cu-**16** in target-positive and negative cells. For [^64^Cu]Cu-**14**, cell uptake plateaued after 60 min incubation (Additional file 1: Supplementary Figure S27). In contrast [^64^Cu]Cu-**16** appeared to approach a plateau only between 120 and 240 min after incubation. This can at least partially be attributed to a higher nonspecific binding as plastic retention was ~ 10 times higher (0.43%–0.59% of tracer stock activity, increasing over time) for [^64^Cu]Cu-**16** as for [^64^Cu]Cu-**14** (0.03%, stable over time). Nevertheless, uptake/binding to PC3 PD-L1-positive cells was significantly different from PC3 mock cells for both compounds. Uptake/binding to PC3 mock cells was indistinguishable from levels in PC3 PD-L1 positive and PC3 mock cells incubated with an excess of unlabeled competitor (BMS-1166, see Additional file 1: Supplementary material 4.1 for details).

### In-vivo studies

For PET imaging, mice were subcutaneously injected with PD-L1 negative cells (PC3-cells carrying a mock construct) on the left and PD-L1 positive cells (PD-L1 PC3-cells) on the right thigh. Imaging was performed at a tumor size of 7 mm and above. After intravenous injection of the radioligand, PET images were acquired at 0–2, 4–5 and 24–25 h p.i. (*n* = 2 for each compound). Additionally, for compounds [^64^Cu]Cu-**14**–[^64^Cu]Cu-**16,** blocking experiments with 500 nmol of the respective, unlabeled compound were performed (injection 5 min prior radiotracer, *n* = 2 each). Due to the low yield of the dimeric PD-L1 radioligand [^64^Cu]Cu-**17**, blocking experiments were not performed. Standardized uptake values of all radioligands and all time points are reported in Additional file 1: Table S1.

**Fig. 2:**
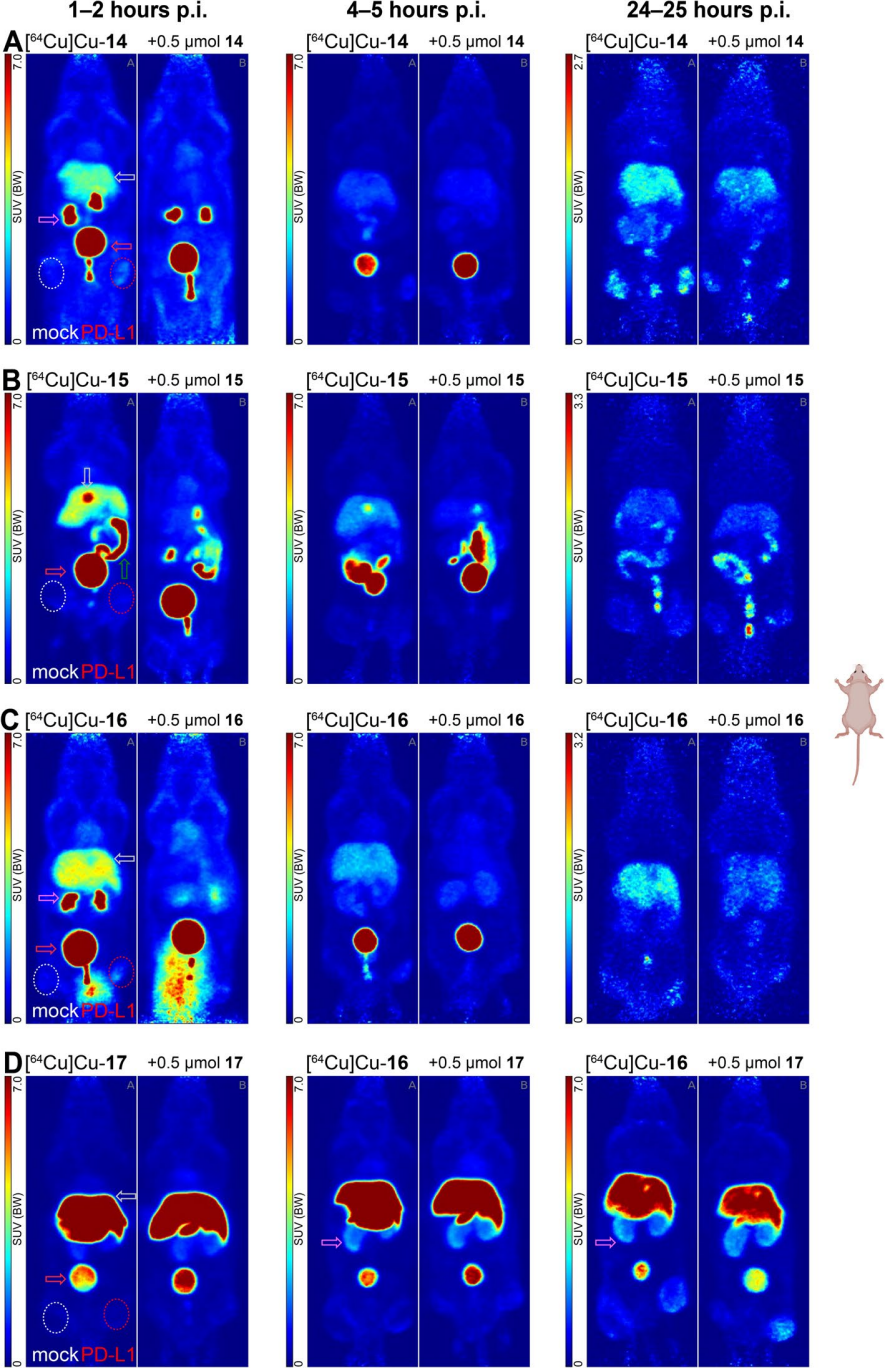
PET images (maximum intensity projections, prone view) of [^64^Cu]Cu-**14**–**17** in tumor-bearing (PC3 + mock left thigh, PC3 + PD-L1 overexpressing right thigh) NMRI-Foxn1 nu/nu mice, at 1–2, 4–5 and 24–25 h p. i.. Scaling at 24–25 h p.i. differs from earlier timepoints. Arrow indicators are color-coded with purple: kidney, silver: liver/gall bladder, red: bladder, green: intestine

In general, the pharmacokinetic profile of all four radioligands was found to differ strongly, depending on the conjugated chelator (see Fig. 2). In this series of compounds, [^64^Cu]Cu-**14** shows the most promising excretion pattern with a favorable renal clearance (SUV_max_ = 22.8 ± 3.0, kidneys, 1–2 h p.i.) and a moderate liver uptake (SUV_max_ = 3.41 ± 0.19). The low accumulation in kidneys (SUV_max_ = 0.72 ± 0.24) and liver (SUV_max_ = 1.54 ± 0.19) at the 4–5 h timepoint indicates a fast clearance of the compound within the first two hours. In accordance with the fast excretion, the uptake in the PD-L1 positive tumor peaked during the first timepoint. SUV_max_ was 1.71 ± 0.04, 99% higher than that of the mock-tumor (SUV_max_ = 0.86 ± 0.16). Under blocking conditions at 1–2 h p.i. and, the liver uptake is reduced by 63% (SUV_max_ = 1.25 ± 0.13 vs SUV_max_ = 3.41 ± 0.19) and the accumulation in the PD-L1 positive tumor is lowered by 13% (SUV_max_ = 1.48 ± 0.44).

For the radioligand [^64^Cu]Cu-**15** with the substituted CB-TE2A-chelator, a hepatobiliary excretion pattern is observed (see Fig. 2). While liver accumulation (SUV_max_ = 3.80 ± 0.33) was in a similar range as observed for [^64^Cu]Cu-**14**, this was contrasted by a low kidney uptake (SUV_max_ = 3.26 ± 0.98) and additional radioactivity in gall bladder and intestines. This is supported by intestinal tracer accumulation at the 4–5 h timepoint. PD-L1 tumor uptake is low at all timepoints (SUV_max_ = 1.03 ± 0.23, 0.66 ± 0.16 and 0.90 ± 0.09 at 1–2, 4, and 24 h p.i., respectively) and was not significantly reduced by preinjection of 500 nmol of cold ligand.

Similar to [^64^Cu]Cu-**14**, the monomeric DiAmSar-conjugate [^64^Cu]Cu-**16** shows clearance via the renal pathway albeit slightly lower (SUV_max_ = 16.2 ± 15.5, 1–2 h p.i.) (see Fig. 2). Consequently, this lower kidney uptake was accompanied by an increased liver accumulation, observable up to 24–25 h p.i. (SUV_max_ = 4.32 ± 0.02 at 1–2 h p.i. to SUV_max_ = 1.77 ± 0.08 at 24 h p.i.). Accumulation in the PD-L1 positive tumor was found to be highest at the first timepoint (SUV_max_ = 1.61 ± 0.93), a contrast of 107% over the mock tumor (SUV_max_ = 0.78 ± 0.36). At the same timepoint and under blocking conditions, uptake is reduced only by 10% in the PD-L1 positive tumor (SUV_max_ = 1.45 ± 0.06). However, liver uptake is reduced by 74%, indicated by SUV_max_ = 1.13 ± 0.04 at the earliest timepoint.

The dimeric DiAmSar-conjugated radioligand [^64^Cu]Cu-**17** shows a high accumulation in the liver during the 1–2 h timepoint (SUV_max_ = 17.0 ± 1.48), which remains high at 4–5 h p.i. (SUV_max_ = 14.4 ± 1.26) and 24–25 h p.i. (SUV_max_ = 8.62 ± 0.84) (see Fig. 2). The absence of accumulated radioactivity in the intestines and the increasing uptake in the kidneys (SUV_max_ = 1.32 ± 0.47, 2.18 ± 0.80 and 2.40 ± 0.60 at 1, 4, 24 h p.i., respectively) indicates rather renal than hepatobiliary clearance and slow excretion of the radioligand [^64^Cu]Cu-**17** over time. This is further supported by the slowly increasing accumulation in the PD-L1 positive tumor over time (from SUV_max_ = 0.49 ± 0.11 at 1–2 h p.i. to SUV_max_ = 2.47 ± 1.04 at 24 h p.i.). These findings support long circulation times and an initial trapping of the radioligand in the liver and its slow release back into the blood pool. The contrast to the PD-L1 negative tumor is increasing over time and is highest (+ 109%) at the 24–25 h p.i. timepoint.

## Discussion

The breakthrough discovery of immune checkpoint inhibitors, e.g. for PD-1 or PD-L1, has significantly advanced cancer therapy. While the administration of such inhibitors reactivates the local immune response in the TME, only about one-third of patients experience a significant treatment response. Currently, decisions on antibody-based checkpoint inhibitor therapy rely on biopsies, which are burdensome. Noninvasive imaging techniques like PET or SPECT enable a minimally invasive whole-body monitoring for predicting and tracking therapies. Among them, small molecule tracers show significant promise due to their short circulation times and high tissue and tumor penetration.

The design of PD-L1 radioligands builds upon our previously described work (Krutzek et al. 2023a), in which we observed remarkably long circulation times for small molecule-based PD-L1 radiotracers. We attributed this phenomenon to albumin binding, likely due to the pincer-like geminal bis(sulfonic acid) moiety. To overcome this challenge, we strategically relocated the sulfonic groups in these radioligands, increasing their spatial separation. Additionally, we introduced a third hydrophilic moiety in the linker structure to facilitate renal clearance (Krutzek et al. 2023b). The synthesis of the PD-L1 ligands reported here, was performed according to our previously developed convergent synthetic route. The intermediate **13** bearing a primary amine served as key building block for the conjugation of different copper-chelators, in order to modulate the hydrophilicity and the net charge of the radioligand. The influence of the net charge has been previously investigated by Raheem et al*.* for DOTA-TATE derivatives. Their work demonstrated that the overall charge of the radioligand not only influences its pharmacokinetic profile but also affects uptake in the target tumor (Raheem et al. 2023; Boswell et al. 2010; Kwon et al. 2022). Consequently, we investigated whether different chelators could improve the in vivo performance of our biphenyl-based PD-L1 radioligands.

Therefore, we selected the chelators NODA-GA, CB-TE2A and DiAmSar, owing to their known excellence in binding copper. In the synthesis of ligand **16**, we obtained a side product, identified as dimeric DiAmSar-structure **17** and subsequently isolated in small quantities. The radiolabeling with ^64^Cu was performed according to established procedures. Proteolytic stability studies showed the highest kinetic stability for the NODA-GA-conjugate [^64^Cu]Cu-**14**. Nevertheless, considering that all radiotracers maintained their integrity at levels surpassing 93% by the 24-h timepoint, the marginally lower kinetic stability observed in the other radioligands can be neglected.

Depending on the type of assay, we observed suitable affinities towards human PD-L1. Using a real-time binding assay, *K*_D_ values were found to be in the low two-digit nanomolar range, similar to our previously reported biphenyl-based PD-L1 ligands. However, in the case of the dimeric DiAmSar-derivative [^64^Cu]Cu-**17**, we were unable to determine the *K*_D_ due to strong adherence to the plastic of petri dishes. We attribute this behavior to the deeply hydrophobic core structure bearing two heavy aromatic binding motifs. Cell uptake studies in target-positive and negative PC3 cells once more confirmed binding specificity, with uptake/binding being significantly higher for PD-L1 overexpressing cells and levels in PC3 mock cells indistinguishable from those incubated with an excess of unlabeled competitor.

PET imaging in tumor bearing mice showed different pharmacokinetics for all four radioligands [^64^Cu]Cu-**14**–[^64^Cu]Cu-**17**. The NODA-GA-conjugate radioligand [^64^Cu]Cu-**14** exhibited the most suitable pattern, with renal clearance and moderate liver uptake. This finding correlates with the determined log *D*_7.4_ value of –3.00 being the lowest in this set of compounds. The substitution with the cyclam-based chelator CB-TE2A ([^64^Cu]Cu-**15**) increased the log *D*_7.4_ by one order of magnitude, which was also reflected in the shift towards a hepatobiliary clearance. The monomeric DiAmSar-conjugate [^64^Cu]Cu-**16** showed renal clearance, however, with an increased liver uptake. Dimerization is a promising concept to increase binding affinities (Foreman 2017), however, in case of our radioligands, the dimer [^64^Cu]Cu-**17** showed a high liver uptake up until 24 h post injection. A determination of the log *D*_7.4_ value and of binding affinities with both the saturation binding assay and the real-time radioligand binding assay was difficult due to the high adherence to plastic surfaces, likely caused by the higher number of aromatic rings. Despite the hydrophobic structure, in vivo PET did not indicate hepatobiliary clearance due to the lack of intestinal tracer uptake even after 24 h. The increasing uptake in the PD-L1 positive tumor suggests trapping of the radioligand in the liver and gradual release into the bloodstream, resulting in the delayed uptake observed in the PD-L1 positive tumor at the final timepoint. This hypothesis is further substantiated by a delayed kidney uptake especially evident at the later timepoints. Overall, the net charge of the four radioligands did not have any discernable impact on the pharmacokinetic behavior. Instead, it was likely dominated by the lipophilicity of the molecules. However, it is worth noting that the accumulation of the four radiotracers in the PD-L1 positive tumor remains low across all timepoints. This underscores the necessity for structural modifications to enhance the binding affinity or in vivo pharmacokinetic behavior. It is important to consider that these structural adjustments, encompassing the linker-chelator system and the incorporation of hydrophilic units, are imperative not only for achieving efficient radiolabeling but also for establishing a favorable pharmacokinetic profile.

## Conclusions

In summary, we successfully synthesized four novel PD-L1 ligands using an efficient convergent synthetic route. These ligands were then conjugated with three distinct copper-chelators and radiolabeled with ^64^Cu. Log *D*_7.4_, proteolytic stability and binding affinities were assessed, showing suitable in vitro performance, albeit high nonspecific binding for [^64^Cu]Cu-**16** and [^64^Cu]Cu-**17**. In vivo, the NODA-GA-substituted derivative [^64^Cu]Cu-**14** showed the most promising pharmacokinetic profile, being quickly cleared through the kidneys. All other compounds caused a much more complex in vivo behavior, with hepatobiliary excretion pattern, liver entrapment and slow tumor uptake. However, tumor uptake into PD-L1 expressing and contrast to PD-L1 negative tumors was comparably low. With the insights gained from this study and potential structural modifications to improve the tumor uptake, promising small-molecule based PD-L1 radioligands for whole-body PET imaging can be developed in the future.

## Methods

### General remarks

All manipulations that needed the exclusion of oxygen and moisture were conducted under an argon gas atmosphere using heat-gun dried flasks and the Schlenk technique. Solvents and chemicals were purchased from Acros Organics, abcr GmbH, Sigma-Aldrich Laborchemikalien GmbH, Fisher Scientific and were utilized without any further purification. Deuterated solvents for NMR studies were obtained from Deutero GmbH. The anhydrous solvents DMF and methanol were acquired from Sigma-Aldrich Laborchemikalien GmbH in Sure/Seal™ bottles. Thin-layer chromatography (TLC) analysis was carried out on pre-coated Merck plates (silica gel 60 F254, Art 5715, 0.25 mm), and visualized using UV light. NMR spectroscopy was performed on either an Agilent DD2-400 MHz NMR or an Agilent DD2-600 MHz NMR spectrometer with ProbeOne. Chemical shifts of ^1^H and ^13^C signals were reported in parts per million (ppm) at 25 °C using TMS as the internal standard. Spectra were calibrated to the respective solvent signal. Mass spectrometry (MS) was conducted with the Xevo TQ-S mass spectrometer by using ESI (electrospray ionization). High-resolution mass spectrometry (HR-MS) was performed with the Nanomate Triversa / Q-Exactive HF by using ESI. Analytical reversed-phase HPLC was performed using an Agilent C18 column (Agilent Zorbax 300SB-C18, 100 mm × 4.6 mm) with acetonitrile/water (0.1% TFA each) as the mobile phase. Semi-preparative reversed-phase HPLC separations were conducted on the Knauer Azura system using Zorbax SB C-18 5 μm 80 Å, 9.4 × 250 mm columns as the stationary phase, with acetonitrile/water (0.1% TFA each) as the mobile phase.

### Synthetic procedures

1-(3-Azidopropyl)piperazine (Pretze and Mamat 2013), 5-bromo-2-hydroxy-4-((2-methoxyethoxy)methoxy)benzaldehyde (**2**), 3-(bromomethyl)-5-(methylsulfonyl)pyridine (**3**), (2,2'-dimethyl-3'-(prop-2-yn-1-yloxy)-[1,1'-biphenyl]-3-yl)methanol (**7**) were described earlier (Krutzek et al. 2023a), as well as 5-bromo-4-((2-methoxyethoxy)methoxy)-2-((5-(methylsulfonyl)pyridin-3-yl)methoxy)benzaldehyde (**4**), 5-bromo-4-hydroxy-2-((5-(methylsulfonyl)pyridin-3-yl)methoxy)benzaldehyde (**5**), 5-bromo-4-((2,2'-dimethyl-3'-(prop-2-yn-1-yloxy)-[1,1'-biphenyl]-3-yl)methoxy)-2-((5-(methylsulfonyl)pyridin-3-yl)methoxy)benzaldehyde (**8**) (5-bromo-4-((2,2'-dimethyl-3'-(prop-2-yn-1-yloxy)-[1,1'-biphenyl]-3-yl)methoxy)-2-((5-methylsulfonyl)pyridin-3-yl)methoxy)benzyl)(sulfo)-d-alanine (**9**) (Krutzek et al. 2023b).

DiAmSar was synthesized according to the literature (Bottomley et al. 1994; Mohammadi et al. 2014).

### General procedure for amide bond formation GP-1

The carboxylic acid, base, coupling reagent and HOBt (1.0 equiv.) were dissolved in DMF at room temperature. After stirring for 5 min, the amine (1.05 equiv.) was added, and the reaction mixture was stirred for the corresponding time. The reaction progress was monitored via analytical HPLC (Agilent Zorbax 300 C-18, 5 μm, 4.6 × 150 mm) with 10–95% acetonitrile (0.1% TFA) in water (0.1% TFA) in a linear gradient over 15 min, 1 mL/min, detection at 254 nm) and upon full conversion of the starting material, DMF was removed in vacuo. The residue was redissolved in a 1:1 mixture of H_2_O/MeCN and purified via semi-preparative RP-HPLC. Subsequently, lyophilization provided the amide compound.

#### (R)-2-((5-Bromo-4-((2,2'-dimethyl-3'-(prop-2-yn-1-yloxy)-[1,1'-biphenyl]-3-yl)methoxy)-2-((5-(methylsulfonyl)pyridin-3-yl)methoxy)benzyl)amino)-3-oxo-3-((2-sulfoethyl)amino)propane-1-sulfonic acid (10)

The carboxylic acid **9** (100.0 mg, 127.0 µmol, 1.00 equiv.) was reacted for 3 h with taurine (17.5 mg, 140.2 µmol, 1.10 equiv.) in presence of DIPEA (44.3 µL, 254.1 µmol, 2.00 equiv.), HOBt (18.9 mg, 140.2 µmol, 1.10 equiv) and HATU (53.1 mg, 139.8 µmol, 1.10 equiv.) as coupling reagent according to GP-1. The product **10** (80.4 mg, 88.7 µmol, 70%) was isolated after semi-preparative RP-HPLC (Agilent Zorbax SB C-18 5 µm 80 Å, 9.4 × 250 mm with 35–80% acetonitrile (0.1% TFA) in water (0.1% TFA) in a linear gradient over 45 min, 6 mL/min, *R*_t_ = 7 min) and lyophilization as a colorless powder. *R*_t_ = 10.67 min (system A), purity > 98%. ^1^H-NMR (400 MHz, DMSO-d_6_) *δ* = 9.35 (bs, 1H), 9.12 (s, 1H), 9.07 (m, 1H), 8.98 (s, 1H), 8.76 (t, ^3^*J* = 5.3 Hz, 1H), 8.53 (s, 1H), 7.65 (s, 1H), 7.50–7.52 (m, 1H), 7.20–7.29 (m, 3H), 7.05–7.10 (m, 2H), 6.75 (d, ^3^*J* = 7.5, 1H), 5.43–5.52 (m, 2H), 5.26 (m, 2H), 4.86 (d, ^4^*J* = 2.2 Hz, 2H), 4.17–4.20 (m, 1H), 4.02 (m, 2H), 3.59 (t, ^4^*J* = 2.2 Hz, 1H), 3.32–3.36 (m, 2H), 2.98–3.02 (m, 1H), 2.71–2.77 (m, 1H), 2.62 (t, ^3^*J* = 7.2 Hz, 2H), 2.04 (s, 3H), 1.84 ppm (s, 3H). ^13^C-NMR (101 MHz, DMSO-d_6_) *δ* = 165.3, 157.1, 156.4, 155.4, 153.2, 146.9, 142.3, 141.5, 136.9, 135.5, 134.9, 134.6, 134.5, 134.5, 132.8, 129.3, 127.7, 126.2, 125.5, 124.1, 122.1, 113.4, 110.9, 104.8, 101.7, 99.9, 79.4, 78.2, 69.7, 67.3, 56.5, 55.8, 49.8, 49.7, 44.3, 43.6, 36.3, 36.3, 15.4, 12.8 ppm. MS (ESI^+^): Mass calculated for [M + H]^+^: *m/z* = 894.10, measured: *m/z* = 893.82.

#### (R)-2-((((9H-Fluoren-9-yl)methoxy)carbonyl)amino)-3-(4-(3-azidopropyl)piperazin-1-yl)-3-oxopropane-1-sulfonic acid (11)

Fmoc-l-Cysteic acid (96.0 mg, 245.6 µmol, 1.00 equiv.) was reacted for 16 h with 1-(3-azidopropyl)piperazine (62.2 mg, 368.0 µmol, 1.50 equiv.) in presence of pyridine (63.2 µL, 736.8 µmol, 3.00 equiv.), HOBt (35.7 mg, 270.2 µmol, 1.10 equiv) and HATU (102.6 mg, 270.2 µmol, 1.10 equiv.) as coupling reagent according to GP-1. The product **11** (106.5 mg, 196.5 µmol, 70%) was isolated after semi-preparative RP-HPLC (Agilent Zorbax SB C-18 5 µm 80 Å, 9.4 × 250 mm with 20–60% acetonitrile (0.1% TFA) in water (0.1% TFA) in a linear gradient over 45 min, 6 mL/min, *R*_t_ = 13 min) and lyophilization as a colorless powder. *R*_t_ = 9.12 min (system A), purity: 98.1%. ^1^H NMR (400 MHz, DMSO-d_6_) *δ* = 7.89 (d, ^3^*J* = 7.5 Hz, 2H), 7.71 (d, ^3^*J* = 7.4 Hz, 2H), 7.41 (t, ^3^*J* = 7.4 Hz, 2H), 7.31–7.34 (m, 2H), 4.77–4.82 (m, 1H), 4.46–4.50 (m, 1H), 4.18–4.27 (m, 3H), 3.45–3.47 (m, 4H), 3.10–3.17 (m, 2H), 2.92–3.03 (m, 2H), 2.72 (m, 1H), 1.88–1.92 ppm (m, 2H). ^13^C NMR (101 MHz, DMSO-d_6_) *δ* = 170.3, 155.6, 143.8, 143.7, 140.7, 127.6, 127.1, 127.0, 125.4, 125.3, 120.1, 65.8, 53.6, 53.4, 50.5, 4.78, 47.1, 46.6, 43.6, 42.5, 42.1, 23.8, 22.9 ppm. MS (HR-ESI^+^): Exact mass calculated for [M + H]^+^: *m/z* = 543.2021, measured: *m/z* = 543.2025.

#### (R)-2-((4-((3'-((1-(3-(4-((((9H-Fluoren-9-yl)methoxy)carbonyl)(sulfo)-d-alanyl)piperazin-1-yl)propyl)-1H-1,2,3-triazol-4-yl)methoxy)-2,2'-dimethyl-[1,1'-biphenyl]-3-yl)methoxy)-5-bromo-2-((5-(methylsulfonyl)pyridin-3-yl)methoxy)benzyl)amino)-3-oxo-3-((2-sulfoethyl)amino)propane-1-sulfonic acid (12)

The alkyne **10** (69.0 mg, 77.1 µmol, 1.00 equiv.) and azide **11** (46.0 mg, 84.8 µmol, 1.10 equiv.) were dissolved in a 1:1 mixture of H_2_O/t^*t*^BuOH. CuSO_4_ (1.2 mg, 7.7 µmol, 0.10 equiv.), THPTA (6.7 mg, 15.4 µmol, 0.20 equiv.) and sodium ascorbate (15.3 mg, 77.1 µmol, 1.00 equiv.) were added to the reaction solution. After stirring at room temperature for 16 h, complete conversion was observed (Agilent Zorbax 300 C-18, 5 μm, 4.6 × 150 mm with 10–95% acetonitrile (0.1% TFA) in water (0.1% TFA) in a linear gradient over 15 min, 1 mL/min, detection at 254 nm). All volatiles were removed under reduced pressure and the crude product was purified by RP-HPLC (Agilent Zorbax SB C-18 5 µm 80 Å, 9.4 × 250 mm with 35–80% acetonitrile (0.1% TFA) in water (0.1% TFA) in a linear gradient over 45 min, 6 mL/min, *R*_t_ = 7 min). After lyophilization, the triazole **12** (86.1 mg, 59.8 µmol, 78%) was obtained as a colorless powder. *R*_t_ = 10.20 min (system A), purity > 99.9%. ^1^H-NMR (400 MHz, DMSO-d_6_) δ = 9.57 (bs, 1H), 9.34 (bs, 1H), 9.11 (s, 1H), 9.07 (s, 1H), 8.98 (bs, 1H), 8.76 (t, ^3^*J* = 5.3 Hz, 1H), 8.52 (s, 1H), 8.30 (s, 1H), 7.88 (d, ^3^*J* = 7.5 Hz, 2H), 7.79–7.81 (m, 1H), 7.70 (d, ^3^*J* = 7.4 Hz, 2H), 7.64 (m, 1H), 7.49–7.51 (m, 1H), 7.41 (t, ^3^*J* = 7.4 Hz, 2H), 7.17–7.34 (m, 6H), 7.07 (d, ^4^*J* = 7.5 Hz, 1H), 6.73 (d, ^3^*J* = 7.4 Hz, 1H), 5.42–5.51 (m, 2H), 5.24–5.34 (m, 2H), 4.79–4.80 (m, 1H), 4.49 (bs, 3H), 4.19–4.27 (m, 4H), 4.02 (bs, 2H), 3.48 (bs), 3.32–3.35 (m), 3.12 (bs), 2.92–3.02 (m), 2.68–2.78 (m), 2.60–2.64 (m, 3H), 2.27 (bs, 2H), 2.34 (s, 3H), 1.81 ppm (s, 3H). ^13^C-NMR (101 MHz, DMSO-d_6_) *δ* = 174.5, 171.3, 170.3, 165.3, 158.5, 158.1, 157.1, 156.4, 156.2, 155.6, 153.4, 147.1, 143.8, 143.7, 143.2, 142.3, 141.6, 140.7, 136.9, 135.5, 134.8, 134.6, 134.6, 134.5, 132.8, 129.3, 127.8, 127.6, 127.1, 127.0, 126.4, 125.5, 125.4, 125.3, 124.5, 124.0, 121.8, 120.1, 72.4, 69.8, 67.3, 65.8, 61.7, 56.5, 53.3, 50.6, 49.8, 49.7, 47.1, 46.7, 46.6, 44.3, 43.6, 42.7, 36.3, 24.2, 15.4, 12.8 ppm. MS (ESI^+^): Mass calculated for [M + H]^+^: *m/z* = 1436.30, measured: *m/z* = 1436.09.

#### (R)-2-Amino-3-(4-(3-(4-(((3'-((2-bromo-5-((5-(methylsulfonyl)pyridin-3-yl)methoxy)-4-((((R)-1-oxo-3-sulfo-1-((2-sulfoethyl)amino)propan-2-yl)amino)methyl)phenoxy)methyl)-2,2'-dimethyl-[1,1'-biphenyl]-3-yl)oxy)methyl)-1H-1,2,3-triazol-1-yl)propyl)piperazin-1-yl)-3-oxopropane-1-sulfonic acid (13)

**12** (85.0 mg, 59.1 µmol, 1.0 equiv.) was dissolved in abs. DMF and sodium azide (7.7 mg, 118.3 µmol, 2.0 equiv.) was added. The reaction mixture was heated to 50 °C for 3 h. After confirming complete conversion by analytical HPLC (Agilent Zorbax 300 C-18, 5 μm, 4.6 × 150 mm) with 10–95% acetonitrile (0.1% TFA) in water (0.1% TFA) in a linear gradient over 15 min, 1 mL/min, detection at 254 nm), the solvent was removed under reduced pressure. The residue was purified by semi-preparative RP-HPLC (Agilent Zorbax SB C-18 5 µm 80 Å, 9.4 × 250 mm with 25–80% acetonitrile (0.1% TFA) in water (0.1% TFA) in a linear gradient over 45 min, 6 mL/min, *R*_t_ = 9 min) and subsequent lyophilization provided the amine **13** (43.2 mg, 35.4 µmol, 60%). *R*_t_ = 10.09 min (system A), purity > 99.9%. ^1^H-NMR (400 MHz, DMSO-d_6_) δ = 9.74 (bs, 1H), 9.34 (bs, 1H), 9.10 (s, 1H), 9.06 (d, ^4^*J* = 1.9 Hz, 1H), 8.97 (bs, 1H), 8.75 (t, ^3^*J* = 5.2 Hz, 1H), 8.51 (d, ^4^*J* = 1.5 Hz, 1H), 8.30 (s, 1H), 8.09–8.10 (m, 2H), 7.63 (d, ^3^*J* = 4.4 Hz, 1H), 7.49 (t, ^3^*J* = 6.4 Hz, 1H), 7.23–7.28 (m, 2H), 7.17–7.19 (m, 2H), 7.07 (d, ^3^*J* = 7.6 Hz, 1H), 6.74 (d, ^3^*J* = 7.4 Hz, 1H), 5.42–5.51 (m, 2H), 5.26–5.34 (m, 2H), 5.18–5.24 (m, 2H), 4.50 (t, ^3^*J* = 6.8 Hz, 2H), 4.17 (bs), 4.01 (bs), 3.85 (bs), 3.55 (bs), 3.31–3.35 (m), 3.17 (bs), 2.95–3.01 (m), 2.72–2.78 (m), 2.61–2.64 (m, 2H), 2.29 (bs, 2H), 2.03 (s, 3H), 1.81 ppm (s, 3H). ^13^C-NMR (101 MHz, DMSO-d_6_) *δ* = 165.3, 158.1, 157.1, 156.4, 156.4, 156.3, 156.2, 153.5, 147.1, 143.3, 142.3, 141.6, 136.8, 135.5, 134.8, 134.7, 134.5, 132.7, 129.3, 128.0, 126.4, 125.5, 124.4, 124.0, 121.8, 113.3, 113.3, 110.7, 109.6, 101.8, 101.7, 99.9, 69.8, 67.3, 61.8, 56.4, 53.2 50.7, 49.8, 49.7, 46.7, 44.2, 43.6, 36.2, 24.3, 15.4, 15.4, 12.8 ppm. MS (ESI^+^): Mass calculated for [M + H]^+^: *m/z* = 1214.23, measured: *m/z* = 1214.51.

#### (R)-2-((R)-4-(4,7-Bis(2-(tert-butoxy)-2-oxoethyl)-1,4,7-triazonan-1-yl)-5-(tert-butoxy)-5-oxopentanamido)-3-(4-(3-(4-(((3'-((2-bromo-5-((5-(methylsulfonyl)pyridin-3-yl)methoxy)-4-((((R)-1-oxo-3-sulfo-1-((2-sulfoethyl)amino)propan-2-yl)amino)methyl)phenoxy)methyl)-2,2'-dimethyl-[1,1'-biphenyl]-3-yl)oxy)methyl)-1H-1,2,3-triazol-1-yl)propyl)piperazin-1-yl)-3-oxopropane-1-sulfonic acid (14a)

(^*t*^Bu_3_)-NODA-GA (2.5 mg, 4.5 µmol, 1.10 equiv.) was reacted for 3 h with amine **13** (5.0 mg, 4.1 µmol, 1.00 equiv.) in presence of DIPEA (1.4 µL, 8.2 µmol, 2.00 equiv.), HOBt (0.6 mg, 4.5 µmol, 1.10 equiv.) and HATU (1.7 mg, 4.5 µmol, 1.10 equiv.) as coupling reagent according to GP-1. The protected NODA-GA derivative **14a** (3.3 mg, 1.9 µmol, 46%) was isolated by semi-preparative RP-HPLC (Agilent Zorbax SB C-18 5 µm 80 Å, 9.4 × 250 mm with 42–80% acetonitrile (0.1% TFA) in water (0.1% TFA) in a linear gradient over 45 min, 6 mL/min, *R*_t_ = 5 min) and lyophilization as a colorless powder. *R*_t_ = 10.07 min (system A), purity > 99.9%. MS (ESI^+^): Mass calculated for [M + H]^+^: *m/z* = 1739.57, measured: *m/z* = 1740.46.

#### 2,2'-(7-((R)-4-(((R)-1-(4-(3-(4-(((3'-((2-bromo-5-((5-(methylsulfonyl)pyridin-3-yl)methoxy)-4-((((R)-1-oxo-3-sulfo-1-((2-sulfoethyl)amino)propan-2-yl)amino)methyl)phenoxy)methyl)-2,2'-dimethyl-[1,1'-biphenyl]-3-yl)oxy)methyl)-1H-1,2,3-triazol-1-yl)propyl)piperazin-1-yl)-1-oxo-3-sulfopropan-2-yl)amino)-1-carboxy-4-oxobutyl)-1,4,7-triazonane-1,4-diyl)diacetic acid (14)

The protected NODA-GA derivative **14a** (3.3 mg, 1.9 µmol, 1.00 equiv.) was stirred in 300 µL of the deprotection cocktail (TFA:DCM:TES:H_2_O, 20:20:8:7, *v*/*v*) at room temperature for 40 h. Complete deprotection was confirmed with analytical RP-HPLC (Agilent Zorbax 300 C-18, 5 μm, 4.6 × 150 mm with 10–95% acetonitrile (0.1% TFA) in water (0.1% TFA) in a linear gradient over 15 min, 1 mL/min, detection at 254 nm). The solvent was removed in vacuo, the residue was purified by semi-preparative RP-HPLC (Agilent Zorbax SB C-18 5 µm 80 Å, 9.4 × 250 mm with 25–80% acetonitrile (0.1% TFA) in water (0.1% TFA) in a linear gradient over 45 min, 6 mL/min, *R*_t_ = 7 min) and after lyophilization, **14** (1.3 mg, 0.8 µmol, 44%) was obtained as a colorless powder. *R*_t_ = 8.98 min (system A), purity > 99.9%. HR-MS (ESI^+^): Exact mass calculated for [M + H]^+^: *m/z* = 1573.3838, measured: *m/z* = 1573.3819.

#### 2-(11-(2-(((R)-1-(4-(3-(4-(((3'-((2-Chloro-4-((((R)-1-((2-(diethoxyphosphoryl)ethyl)amino)-1-oxo-3-sulfopropan-2-yl)amino)methyl)-5-((5-(methylsulfonyl)pyridin-3-yl)methoxy)phenoxy)methyl)-2,2'-dimethyl-[1,1'-biphenyl]-3-yl)oxy)methyl)-1H-1,2,3-triazol-1-yl)propyl)piperazin-1-yl)-1-oxo-3-sulfopropan-2-yl)amino)-2-oxoethyl)-1,4,8,11-tetraazabicyclo[6.6.2]hexadecan-4-yl)acetic acid (15)

CB-TE2A (4.2 mg, 12.3 µmol, 3.00 equiv.) was reacted for 5 h with the amine **13** (5.0 mg, 4.1 µmol, 1.00 equiv.) in presence of DIPEA (1.4 µL, 3.5 µmol, 2.00 equiv.) and HOBt (0.6 mg, 4.5 µmol, 1.10 equiv.) and HATU (1.6 mg, 4.1 µmol, 1.00 equiv.) as coupling reagent according to GP-1. The product **15** (5.4 mg, 3.5 µmol, 85%) was isolated by semi-preparative RP-HPLC (Agilent Zorbax SB C-18 5 µm 80 Å, 9.4 × 250 mm with 30–80% acetonitrile (0.1% TFA) in water (0.1% TFA) in a linear gradient over 45 min, 6 mL/min, *R*_t_ = 9 min) and lyophilization as a colorless powder. *R*_t_ = 8.77 min (system A), purity > 99.9%. HR-MS (ESI^+^): Exact mass calculated for [M/2 + H]^+^: *m/z* = 770.2221, measured: *m/z* = 770.2288.

#### 5-(((R)-1-(4-(3-(4-(((3'-((2-bromo-5-((5-(methylsulfonyl)pyridin-3-yl)methoxy)-4-((((R)-1-oxo-3-sulfo-1-((2-sulfoethyl)amino)propan-2-yl)amino)methyl)phenoxy)methyl)-2,2'-dimethyl-[1,1'-biphenyl]-3-yl)oxy)methyl)-1H-1,2,3-triazol-1-yl)propyl)piperazin-1-yl)-1-oxo-3-sulfopropan-2-yl)amino)-5-oxopentanoic acid (16a)

The amine **13** (21.5 mg, 17.7 µmol, 1.00 equiv.), glutaric anhydride (10.1 mg, 88.5 µmol, 5.00 equiv.) and abs. DIPEA (6.0 µL, 35.4 µmol, 2.00 equiv.) were dissolved in abs. DMF (500 µL). The reaction mixture was stirred for 16 h and complete conversion of the starting material was confirmed via analytical RP-HPLC (Agilent Zorbax 300 C-18, 5 μm, 4.6 × 150 mm with 10–95% acetonitrile (0.1% TFA) in water (0.1% TFA) in a linear gradient over 15 min, 1 mL/min, detection at 254 nm). The solvent was removed, and the residue purified by semi-preparative RP-HPLC (Agilent Zorbax SB C-18 5 µm 80 Å, 9.4 × 250 mm with 25–80% acetonitrile (0.1% TFA) in water (0.1% TFA) in a linear gradient over 45 min, 6 mL/min, *R*_t_ = 8 min). After lyophilization, the carboxylic acid **16a** (19.8 mg, 14.9 µmol, 84%) was obtained as a colorless solid. *R*_t_ = 8.73 min (system A), purity > 99.9%. ^1^H-NMR (400 MHz, DMSO-d_6_) δ = 9.54 (bs, 1H), 9.34 (bs, 1H), 9.12 (s, 1H), 9.07 (d, ^4^*J* = 2.0 Hz, 1H), 8.97 (bs, 1H), 8.76 (t, ^3^*J* = 5.5 Hz, 1H), 8.52 (s, 1H), 8.30 (s, 1H), 8.24 (t, ^3^*J* = 7.0 Hz, 1H), 7.65 (s, 1H), 7.49–7.51 (m, 1H), 7.17–7.29 (m, 3H), 7.07 (d, ^3^*J* = 7.6 Hz, 1H), 6.73 (d, ^3^*J* = 7.4 Hz, 1H), 5.42–5.51 (m, 2H), 5.18–5.34 (m, 4H), 5.01 (bs, 1H), 4.48–4.51 (m), 4.17 (bs), 3.99–4.02 (m), 3.32–3.49 (m), 2.92–3.11 (m), 2.71–2.77 (m, 1H), 2.61–2.64 (m, 2H), 2.27 (bs, 2H), 2.16–2.20 (m, 2H), 2.04–2.09 (m, 4H), 1.81 (s, 3H), 1.64–1.68 ppm (2H). ^13^C-NMR (101 MHz, DMSO-d_6_) *δ* = 174.2, 171.2, 170.2, 165.3, 157.1, 156.4, 156.2, 153.4, 147.0, 143.2, 142.3, 141.6, 136.9, 134.9, 134.6, 134.6, 134.5, 132.8, 129.3, 129.3, 127.8, 126.4, 125.5, 124.5, 124.0, 121.8, 113.4, 110.7, 109.6, 101.7, 99.9, 69.8, 67.3, 61.7, 60.4, 56.5, 53.9, 53.6, 50.5, 49.8, 49.7, 46.7, 45.0, 44.7, 44.3, 43.6, 43.4, 42.7, 36.2, 33.9, 32.9, 24.3, 20.5, 15.4, 12.9 ppm. MS (ESI^+^): Mass calculated for [M + H]^+^: *m/z* = 1328.26, measured: *m/z* = 1328.16.

#### (R)-2-(5-((8-amino-3,6,10,13,16,19-hexaazabicyclo[6.6.6]icosan-1-yl)amino)-5-oxopentanamido)-3-(4-(3-(4-(((3'-((2-bromo-5-((5-(methylsulfonyl)pyridin-3-yl)methoxy)-4-((((R)-1-oxo-3-sulfo-1-((2-sulfoethyl)amino)propan-2-yl)amino)methyl)phenoxy)methyl)-2,2'-dimethyl-[1,1'-biphenyl]-3-yl)oxy)methyl)-1H-1,2,3-triazol-1-yl)propyl)piperazin-1-yl)-3-oxopropane-1-sulfonic acid (16) and dimeric DiAmSar-Ligand (17)

The carboxylic acid **16a** (6.00 mg, 4.5 µmol, 1.00 equiv.) was reacted for 3 h with DiAmSar (1.4 mg, 4.5 µmol, 1.00 equiv.) in presence of DIPEA (1.5 µL, 9.0 µmol, 2.00 equiv.), HOBt (0.6 mg, 4.72 µmol, 1.05 equiv) and HATU (1.8 mg, 4.7 µmol, 1.05 equiv.) as coupling reagents according to GP-1. The monomeric ligand **16** (5.7 mg, 3.5 µmol, 77%) and the dimeric ligand **17** (1.1 mg, 0.4 µmol, 9%) were isolated by semi-preparative RP-HPLC (Agilent Zorbax SB C-18 5 µm 80 Å, 9.4 × 250 mm with 25–80% acetonitrile (0.1% TFA) in water (0.1% TFA) in a linear gradient over 45 min, 6 mL/min, *R*_t_ = 8 min (monomer), *R*_t_ = 13 min (dimer)) and lyophilization as a colorless powders. *R*_t_ = 8.73 min (system A), purity > 99.9% (monomer). *R*_t_ = 9.52 min (system A), purity > 99.9% (dimer). HR-MS (ESI +): Exact mass calculated for [M/2 + H] + : *m/z* = 813.7660, measured: *m/z* = 813.7735 (monomer) and exact mass calculated for [M/2 + H] + : *m/z* = 1469.3874, measured: *m/z* = 1469.3961.

### Radiochemistry

For the radiolabeling of the chelator-bearing compounds, 8 nmol of the corresponding radiotracer was used (8 µL of a 1 M solution in DMSO). The production of ^64^Cu was performed in-house through the nuclear reaction ^64^Ni(p,n) → ^64^Cu, with a method previously reported (Thieme et al. 2012). The resulting [^64^Cu]CuCl_2_ was obtained in a solution with a concentration range of 0.1–0.01 M HCl.

In case of the ligands bearing a NODA-GA- (**14**) or CB-TE2A-chelator (**15**), the reaction medium was a 1 M HEPES solution adjusted prior to pH 4.5 with HCl. In case of DiAmSar bearing ligands **16** and **17**, the radiolabeling was conducted in 1 M HEPES solution without adjusting the pH. The ligand was incubated with ^64^Cu at the respective temperature and time (50 °C and 10 min for **14**, 95 °C and 60 min for **15**, r.t. and 15 min for **16** and **17**) while shaking at 300 rpm.

A minor quantity of the resultant mixture was applied onto iTLC-SG chromatography paper, serving as the stationary phase. The paper was then subjected to the mobile phase, consisting of a 0.1 M citrate solution (pH 4, adjusted using 1 M NaOH). Analysis of the chromatography paper was performed with a radioisotope thin layer analyzer (Rita Star, Elysia-Raytest GmbH, Straubenhardt). The efficiency of the labeling process was determined by calculating the ratio between unbound radiometal (*R*_f_ = 0.9) and radio-metal complex (*R*_f_ = 0), accomplished through integration of their respective areas. Labeling efficiencies exceeding 95% were attained for all radiotracers.

### Log D_7.4_ determination

The *n*-octanol/water distribution coefficients for each radioligand were determined using the shake-flask method. Each experiment was conducted in triplicate. To initiate the experiment, 30 µL of the labeling mixture was combined with 570 µL of PBS (pH 7.4) and 600 µL of *n*-octanol in a 1.5 mL Eppendorf-Tube. The resulting mixture was shaken at room temperature at 1500 rpm for 5 min before undergoing centrifugation. An aliquot was extracted from each phase, and radioactivity was measured on a γ-counter (ISOMED 2160). Subsequently, mean values were calculated and adjusted for background activity. The log *D*_7.4_ value was determined utilizing the equation:

$$\log D_{7.4} = {\text{log}}\left( {\frac{{A_{n - octanol} }}{{A_{{PBS{ }}} }}} \right)$$

### Proteolytic stability studies

For proteolytic stability studies, a solution of the radiolabeled compound was diluted with human serum in a ratio of 1:10. This mixture then underwent incubation at 37 °C while being shaken at 300 rpm. At each designated timepoint (1, 24 and 48 h), an aliquot was withdrawn and added to a detergent solution (water with 20% *v*/*v* EtOH, 5% *v*/*v* 5 mM aqueous EDTA, 0.5% *v*/*v* Triton X-100, 0.1% *m*/*v* saponin, 0.05% *v*/*v* 0.5 mM aqueous *o*-phenanthroline) in a ratio of 1:2 for precipitation of serum proteins. The mixture was cooled on ice for 5 min, followed by centrifugation at 12,000 g for 5 min. A minor portion of the resulting supernatant was subjected to analytical radio-HPLC (reversed-phase, Phenomenex Jupiter 300 C-18, 5 µm, 4.6 × 250 mm) with 10–95% acetonitrile (0.1% TFA) in water (0.1% TFA) in a linear gradient over 15 min, 1 mL/min, γ-detection). The assessment and graphical representations were performed using OriginPro® 9.0.

### Cell lines and cell culture

PC3-PD-L1 along with PC3-mock cells were previously described in detail (Krutzek et al. 2023b). The cells were cultured in RPMI-1640 medium under normoxic conditions (5% CO_2_, 37 °C). Passage of cells was performed at approximately 90% confluency.

For the saturation binding experiments, cells were detached with 0.05% Trypsin–EDTA (Gibco, USA), and counted (CASY1 cell counter, Schaerfe System, Reutlingen, Germany). Cells were then diluted in medium to ~ 160,000 cells/mL and seeded in 48 well plates (Falcon Multiwell #353078, ThermoFisher, Karlsruhe, Germany) for at least 2 days prior to saturation binding experiments. For real-time radioligand binding, approximately 400,000 cells/mL were seeded into one side of petri dishes (3 mL, Nunclon, # 150350, ThermoFisher Germany) ~ 24 h before the experiments.

### Saturation binding assay

For the determination of dissociation constant (*K*_D_) and the number of binding sites (B_max_), saturation binding experiments were conducted in a minimum of three independent experiments, with each data point formed from triplicates. Human PD-L1 transduced PC3 cells, grown under the previously described conditions, were first brought to room temperature, and subsequently cooled on ice (each 15 min). The culture medium was replaced with 200 µL of assay buffer [PBS + 2.5% *w*/*v* bovine serum albumin (BSA; #1ETA, Carl Roth, Karlsruhe, Germany)] for total binding (TB) conditions, or assay buffer containing 300 µM of BMS-1066 (Guzik et al. 2017) (dissolved in DMSO, resulting in 0.03% *v*/*v*) for the assessment of nonspecific binding (NSB).

After a 15-min preincubation for TB/NSB, 200 µL of eight serial 1:1 dilutions (ranging from 500 to 3.91 nM) of the corresponding radioligand were added to the wells. The cells were then incubated on ice for 90 min, followed by removal of the incubation medium and washing with ice-cold assay buffer (3 washes of approximately 1 min each). Subsequently, cell lysis was performed using 500 µL of 0.1 M NaOH + 1% SDS. The radioactivity (measured in counts per minute, CPM) of 400 µL of the lysate was assessed using a gamma counter (Perkin Elmer Wizard 1480). Additionally, binding to polystyrene was determined in 400 µL of lysate, and the activity of stock solutions (50 µL) was measured. All recorded counts were corrected for radioactive decay to a reference time (end of radiolabeling). As BSA increases protein content, an additional plate underwent the same procedures (preincubation, incubation, and washing) using PBS without BSA/radioligand for each experimental set. The protein content of the lysates (from 24 or 48 wells) was quantified using a BCA assay, and the mean value (µg/mL) was employed for this specific dataset (plate). Utilizing the mean protein content and molar activity, final values (pmol/mg protein) were derived from the CPM measurements. Subsequently, the processed data underwent nonlinear iterative curve fitting using GraphPad Prism 9 software, yielding B_max_ (expressed in pmol/mg protein) and *K*_D_ (expressed in nM). For details on cell uptake experiments, please see the Supplementary material (4.1).

### Real-time radioligand binding studies

To investigate binding kinetics, including the association rate constant (*k*_a_) and dissociation rate constant (*k*_d_), as well as the dissociation constant (*K*_D_), a real-time assay system (LigandTracer Yellow or LigandTracer White, Ridgeview Instruments AB, Uppsala, Sweden) was utilized. The petri dish contained attached cells in medium (3 mL) on one side, while radioactivity was detected on the opposing side. The petri dish is set upon an inclined base, allowing continuous measurement of alternating portions of the dish for bound radioligand (cells) compared to the background signal (no cells). Binding experiments were carried out at room temperature using CO_2_-independent medium (Gibco #18045088, ThermoFisher Germany).

The association phase consisted of incubation with two concentrations (30 and 90 nM, each for 90 min) of our ^64^Cu-labeled PD-L1-radioligands, binding to PC3 PD-L1 overexpressing cells. Following this, the incubation medium was replaced with fresh medium, and the dissociation phase was monitored for a minimum of 2 h. This methodology ensured dependable kinetic measurements, as previously demonstrated (Önell and Andersson 2005). Data were recorded as decay-corrected counts per second (CPS). Subsequently, binding data were analyzed using TraceDrawer software (version 1.9.2, Ridgeview Instruments AB, Uppsala, Sweden). Traces were imported, potential spikes (instances of sudden CPS increases exceeding 100% of the previous data point) were eliminated, and each trace was normalized to its own baseline (set as 0%) and its maximum value (set as 100%).

### Animals, biodistribution and PET imaging

Male athymic NMRI-nude mice (Rj:NMRI-Foxn1nu, Janvier Labs, Le Genest-Saint-Isle, France) aged between 8 to 16 weeks were employed for the study. General anesthesia was induced [~ 10% desflurane (Baxter, Deerfield, IL, USA) in 30 vol% oxygen + air] under controlled warming (37°C), and mice were subcutaneously injected with 3–5 × 10^6^ PC3 PD-L1 and PC3 mock cells (in 50 µL PBS + 50 µL Matrigel, Corning, Glendale, CA, USA) into the right and left thigh, respectively. The growth of tumors was systematically monitored three times per week through caliper measurements, and animals with tumor sizes above 7 mm were included in the subsequent experiments.

PET and X-ray computed tomography (CT) scans were conducted under general anesthesia using a small animal nanoScan PET/CT scanner (Mediso, Budapest, Hungary), capable of imaging four animals simultaneously. CT images served attenuation correction and anatomical referencing. A total of three individual scans were conducted, corresponding to the timeframes of 0–2 h (dynamic reconstruction), 4–5 h, and 24–25 h (one frame reconstruction) after injection.

The PD-L1 radiotracer candidates (in 300 µL sterile 0.9% NaCl/HEPES buffer, pH 6–7, containing between 7 and 12 MBq, with molar activities exceeding 12 GBq/µmol) were intravenously delivered over 30 s through a catheter in the lateral tail vein. The PET acquisition was initiated simultaneously with the administration.

The acquired three-dimensional list mode data were sorted and binned within a 400–600 keV energy window, forming 36 time frames (15 × 10 s, 5 × 30 s, 5 × 60 s, 4 × 300 s, 3 × 600 s, 3 × 1200 s). Reconstruction of the time frames was accomplished using the Tera-TomoTM 3D algorithm, utilizing a voxel size of 0.4 mm and applying corrections for decay, scatter, and attenuation. The images were subjected to post-processing and analysis using Rover software (ABX GmbH, Radeberg, Germany), and were depicted as maximum intensity projections (MIPs) at the specified timepoints and scaling.

Three-dimensional volumes of interest were defined using fixed thresholding at 35–40% of the maximal measured intensity. Standardized uptake values (SUV = [MBq detected activity/mL tissue] / [MBq injected activity/g body weight], in mL/g) were then calculated within the selected volumes of interest, encompassing PD-L1 and mock tumors, as well as metabolizing organs.

### Data and statistical analysis

The data are expressed as the mean ± standard deviation. All statistical analyses were conducted using GraphPad Prism, version 9.5 (GraphPad Software Inc., San Diego, CA, USA).

### Supplementary Information

**Additional file 1**. Supplementary material.

## Data Availability

The datasets used and/or analyzed during the current study are available from the corresponding author on reasonable request.

## Abbreviations

abs.: Absolute
BSA: Bovine serum albumin
PET: Positron emission tomography
CB-TE2A: 1,4,8,11-Tetraazabicyclo[6.6.2]hexadecane-4,11-diacetic acid
CuAAC: Copper-catalyzed azide-alkyne cycloaddition
DiAmSar: 1,8-Diamino-3,6,10,13,16,19-hexaazabicyclo[6,6,6]-eicosane
DIPEA: Diisopropylethylamine
DMEAD: Di-2-methoxyethylazodicarboxylate
HATU: Hexafluorophosphate azabenzotriazole tetramethyl uranium
HEPES: (4-(2-Hydroxyethyl)-1-piperazineethanesulfonic acid)
HOBt: 1-Hydroxybenzotriazol
MEM: 2-Methoxyethoxymethyl
NODA-GA: 4-(4,7-Bis(2-(*tert*-butoxy)-2-oxoethyl)-1,4,7-triazacyclononan-1-yl)-5-(*tert*-butoxy)-5-oxopentanoic acid
PD-1: Programmed cell death protein 1
PD-L1: Programmed death ligand 1
SUV: Standard up-take value
TES: Triethylsilane
THPTA: Tris(3-hydroxypropyltriazolylmethyl)amine
