# Supplementary material for: Chelator impact: investigating the pharmacokinetic behavior of copper-64 labeled PD-L1 radioligands
Source: EJNMMI Radiopharm Chem. 2024 Feb 19;9:14. doi: 10.1186/s41181-024-00243-5 (PMC10876507; doi:10.1186/s41181-024-00243-5)
Supplement: Supplementary file 1 — Additional file 1. Supplementary material. [file 41181_2024_243_MOESM1_ESM.pdf]

Chelator impact: investigating the pharmacokinetic behavior of copper-64 labeled PD-L1 radioligands

Fabian Krutzek[1], Cornelius K. Donat[1], Sven Stadlbauer[1,2]*

[1]Helmholtz-Zentrum Dresden-Rossendorf, Institute of Radiopharmaceutical Cancer Research, Bautzner Landstraße 400, D-01328 Dresden, Germany

[2]School of Science, Faculty of Chemistry and Food Chemistry, Technical University Dresden, 01069 Dresden, Germany

Content

1. ^{1}H and ^{13}C NMR spectra of unknown compounds 2
2. HPLC-chromatograms of HPLC purified compounds 6
3. Mass Spectra of literature unknown compounds 9
4. Biological data 14

1. ^{1}H and ^{13}C NMR spectra of unknown compounds

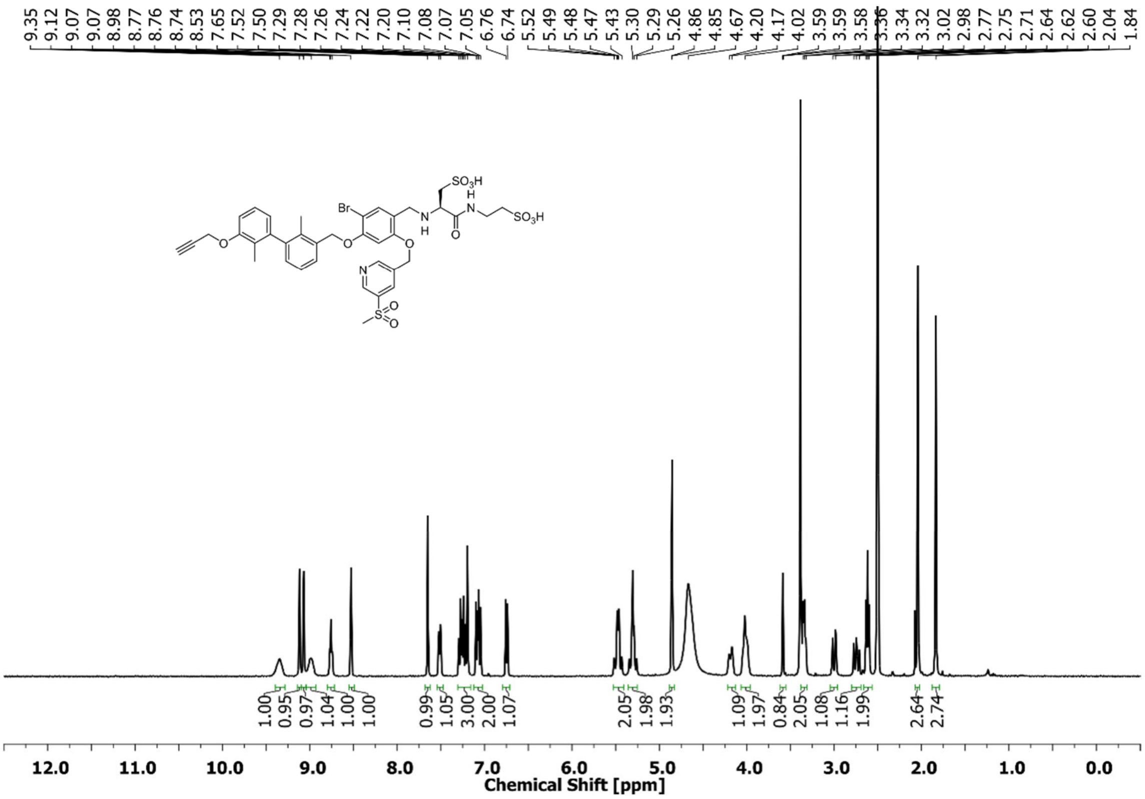

Figure S1: ^{1}H NMR spectrum (DMSO-d_6, 400 MHz, 298 K) of compound **10**.

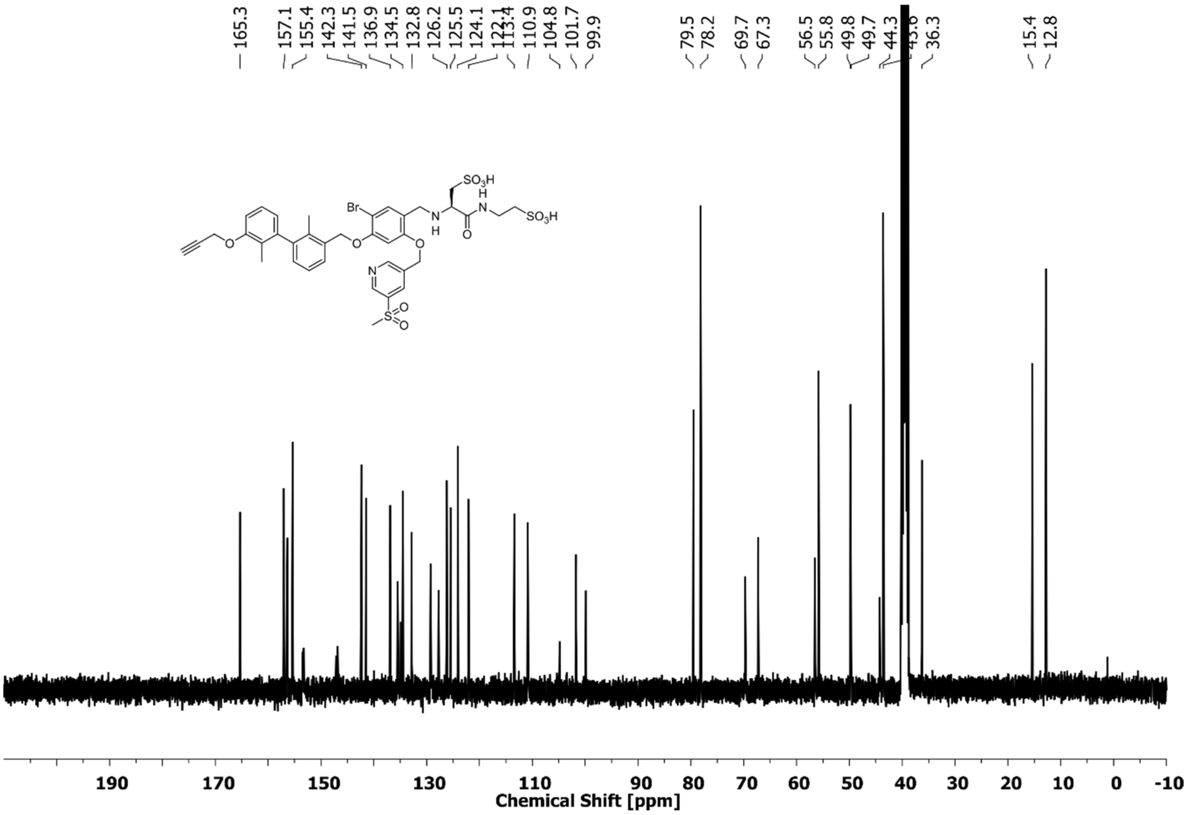

Figure S2: ^{13}C NMR spectrum (DMSO-d_6, 101 MHz, 298 K) of compound **10**.

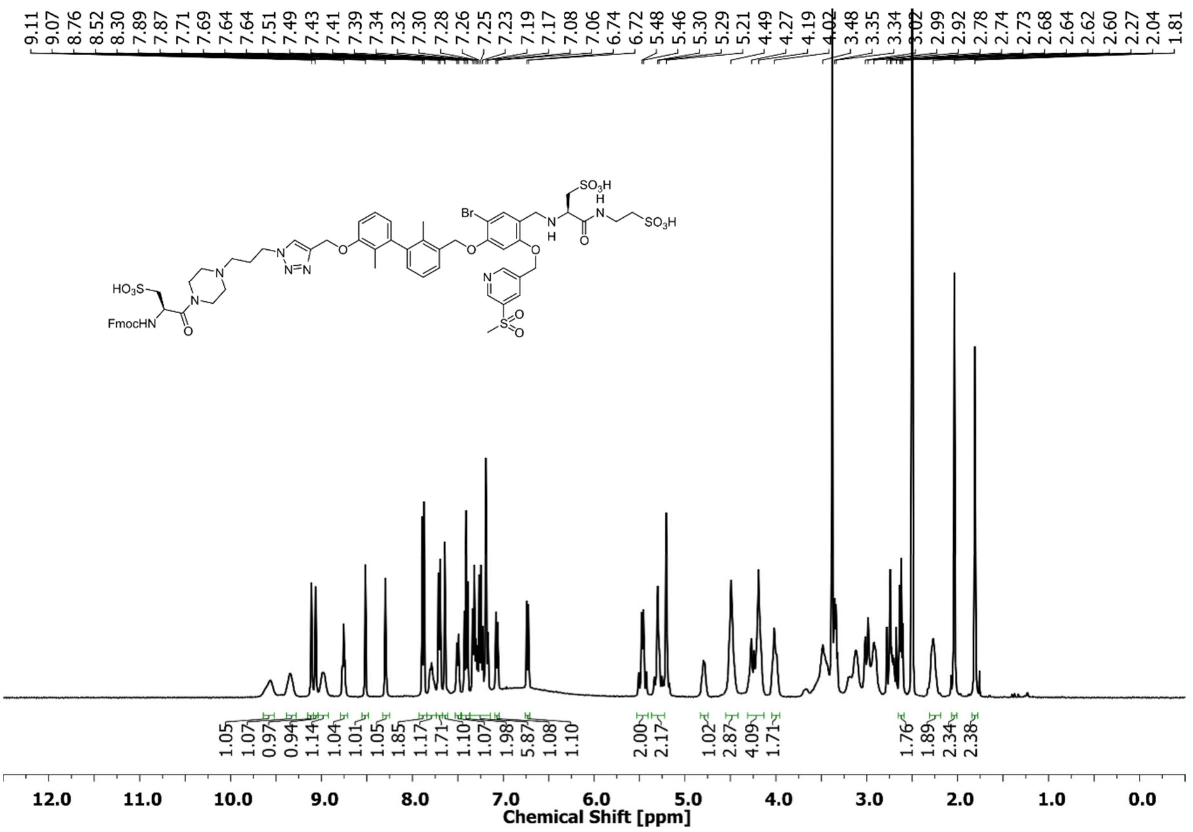

Figure S3: ^{1}H NMR spectrum (DMSO-d_6, 400 MHz, 298 K) of compound **12**.

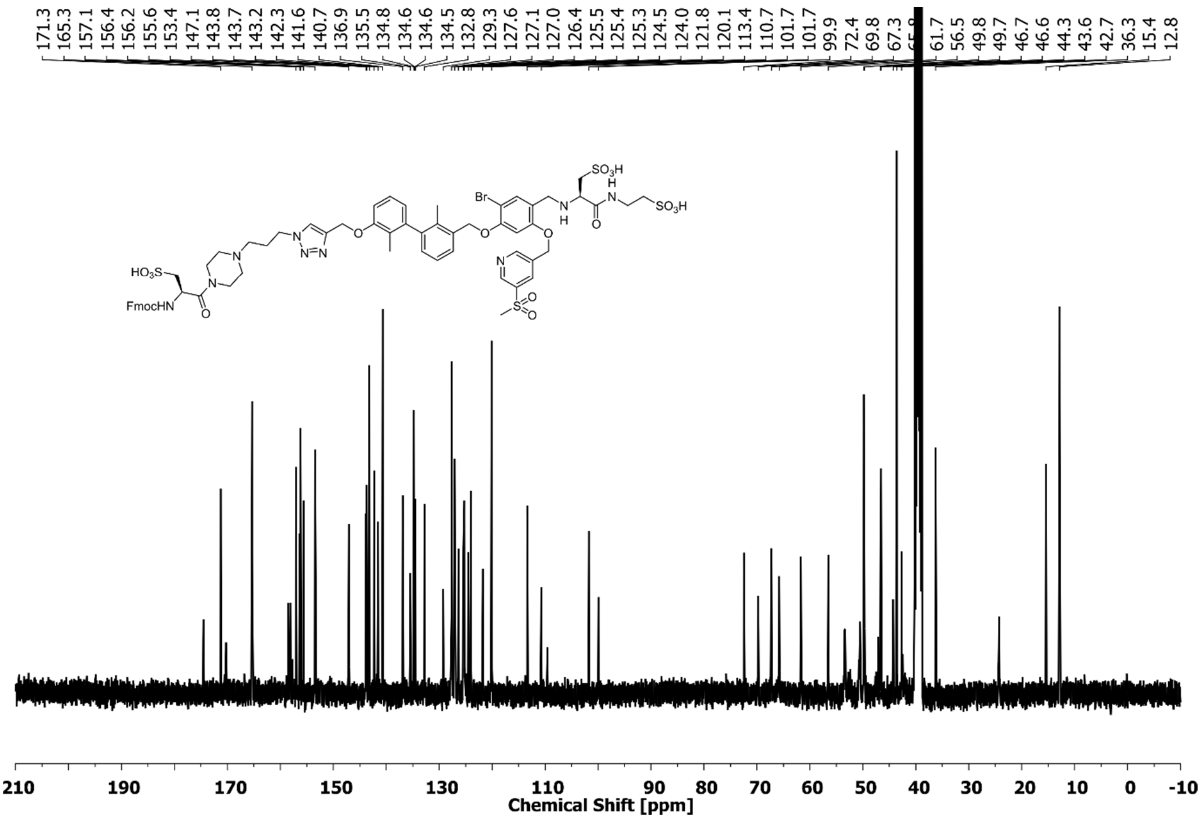

Figure S4: ^{13}C NMR spectrum (DMSO-d_6, 101 MHz, 298 K) of compound **12**.

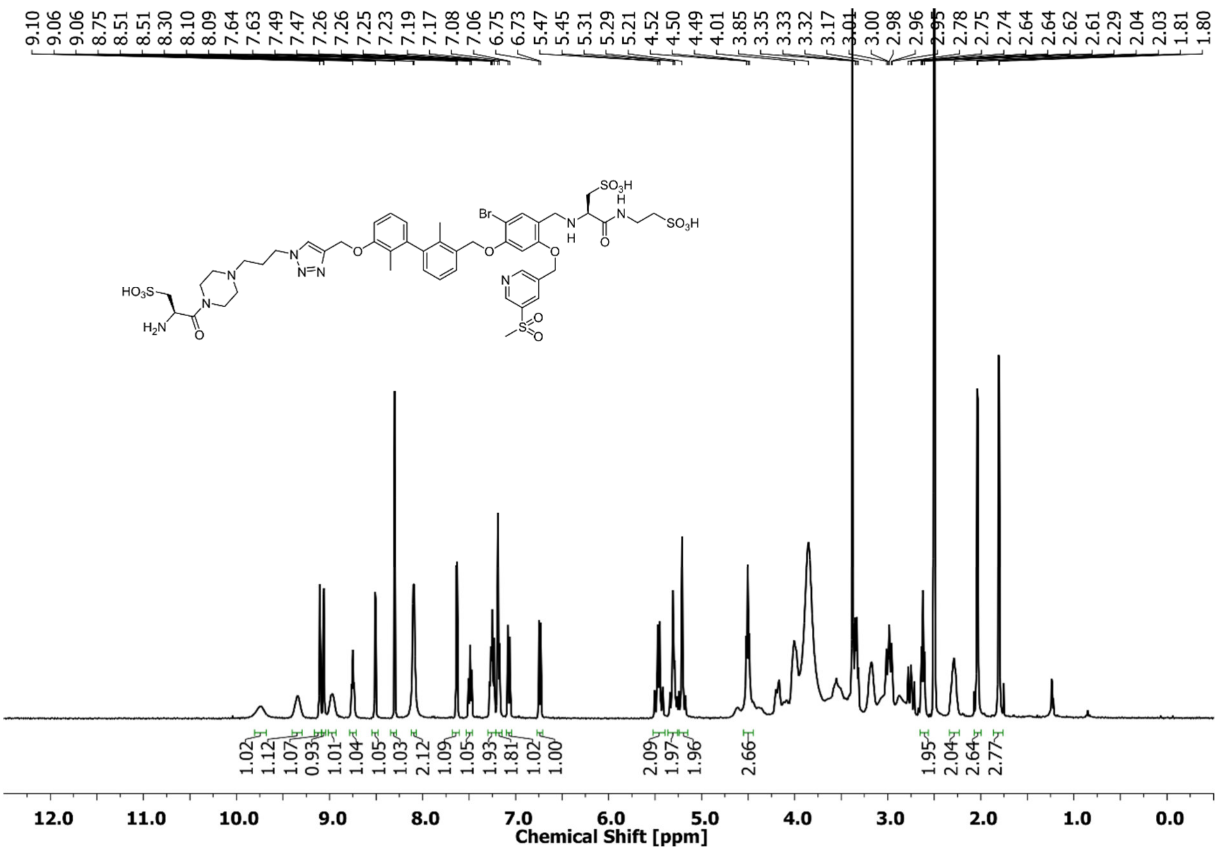

Figure S5: ^{1}H NMR spectrum (DMSO-d_6, 400 MHz, 298 K) of compound **13**.

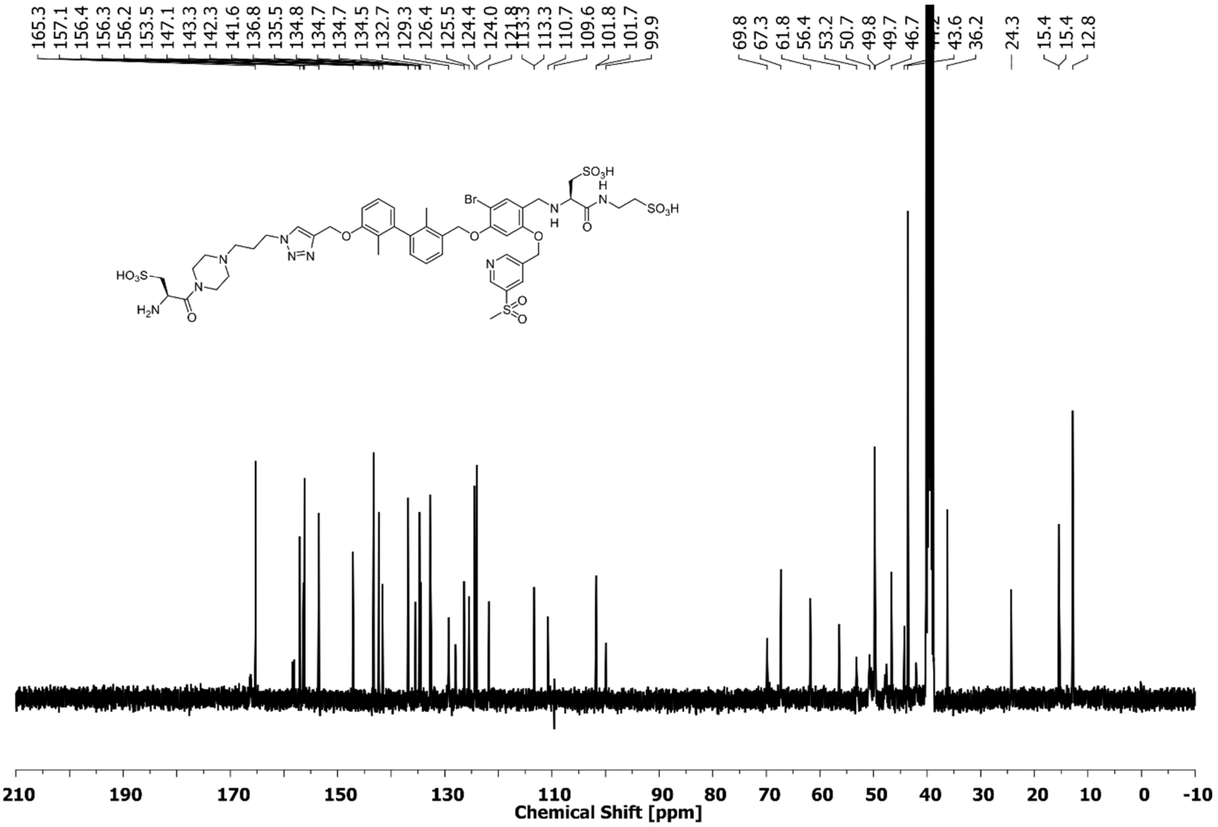

Figure S6: ^{13}C NMR spectrum (DMSO-d_6, 101 MHz, 298 K) of compound **13**.

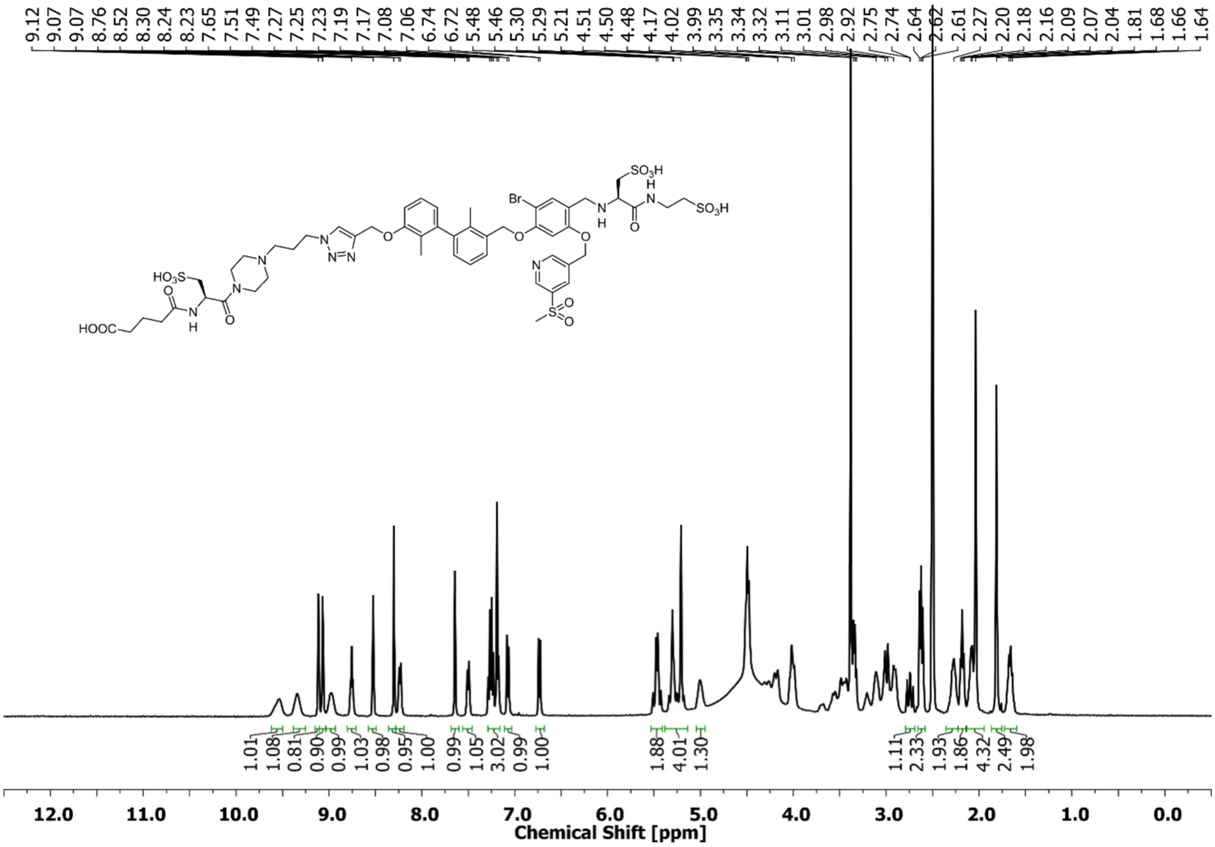

Figure S7: ^{1}H NMR spectrum (DMSO-d_6, 400 MHz, 298 K) of compound **16a**.

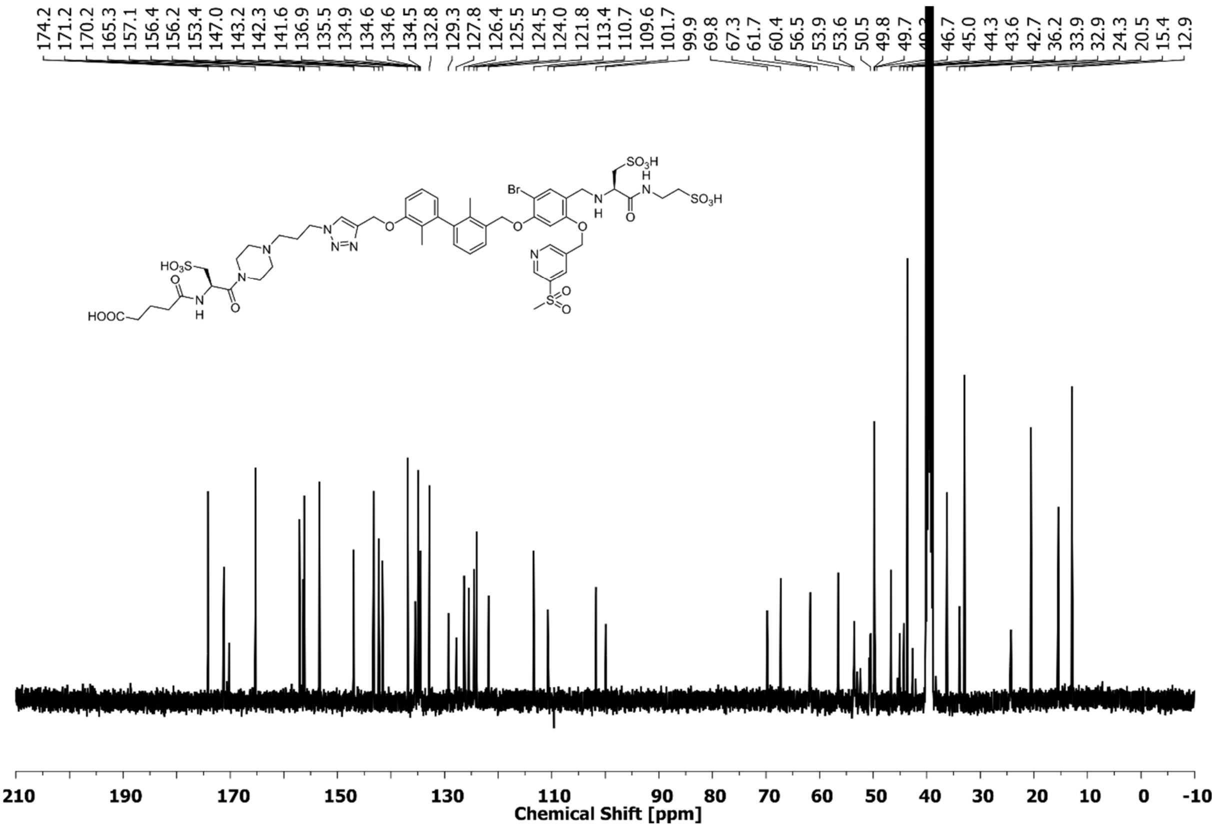

Figure S8: ^{13}C NMR spectrum (DMSO-d_6, 101 MHz, 298 K) of compound **16a**.

2. HPLC-chromatograms of HPLC purified compounds

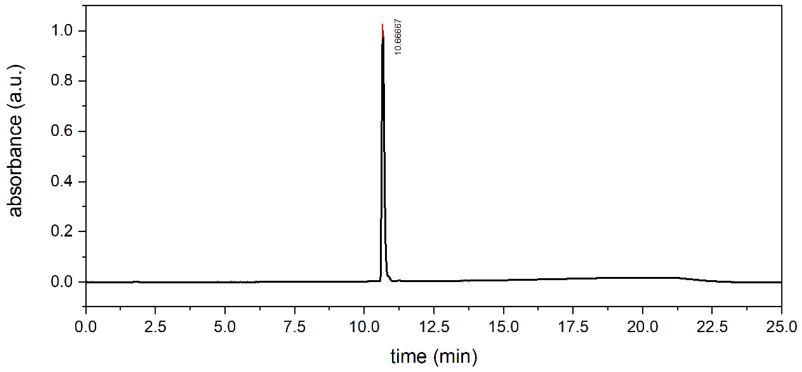

Figure S9: Analytical RP-HPLC chromatogram (System A) of compound **10**.

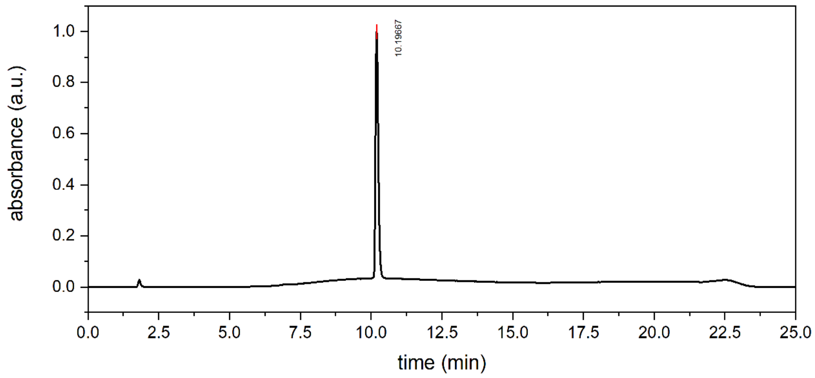

Figure S10: Analytical RP-HPLC chromatogram (System A) of compound **12**.

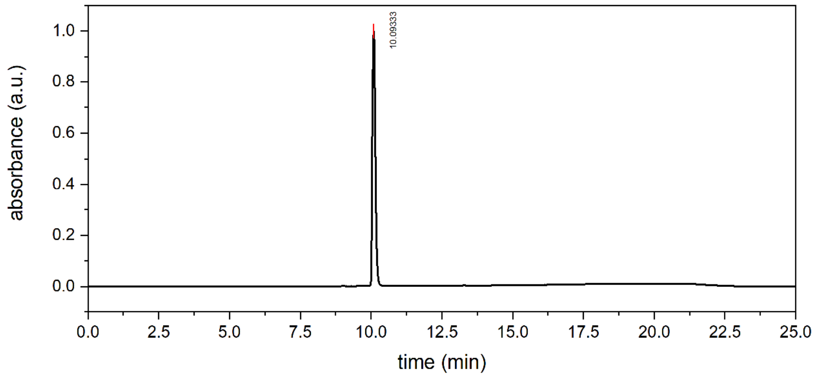

Figure S11: Analytical RP-HPLC chromatogram (System A) of compound **13**.

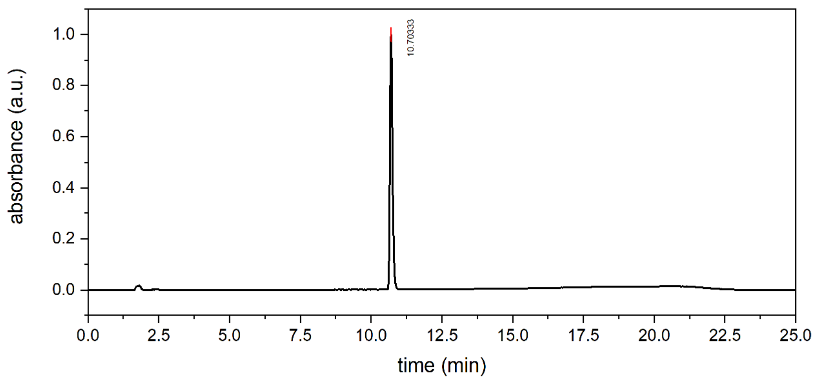

Figure S12: Analytical RP-HPLC chromatogram (System A) of compound **14a**.

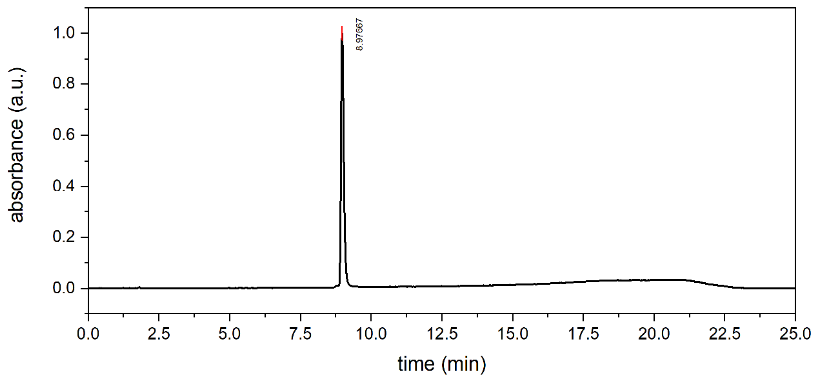

Figure S13: Analytical RP-HPLC chromatogram (System B) of compound **14**.

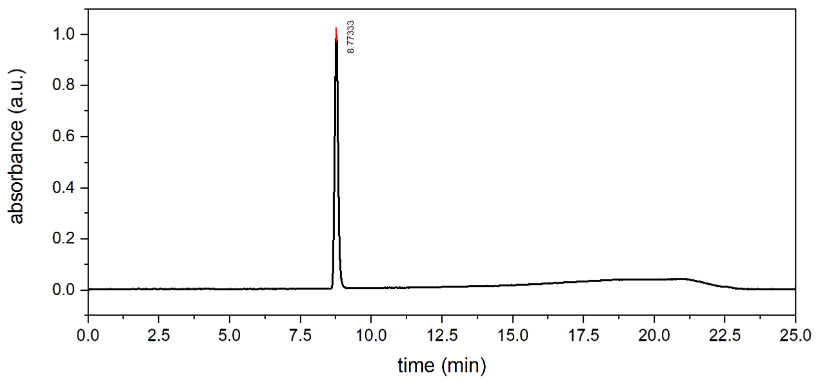

Figure S14: Analytical RP-HPLC chromatogram (System A) of compound **15**.

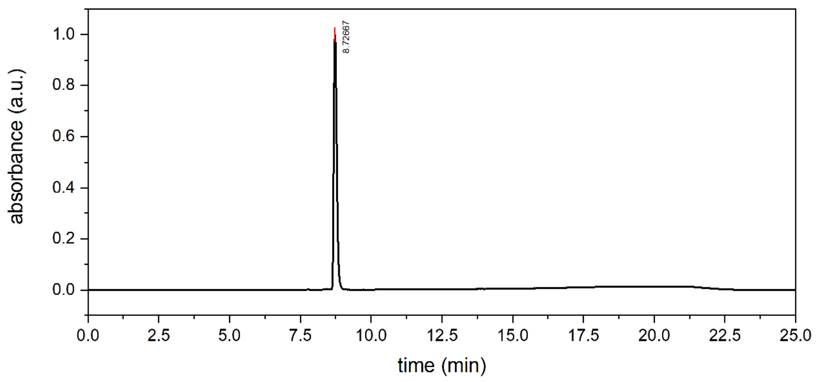

Figure S15: Analytical RP-HPLC chromatogram (System A) of compound **16a**.

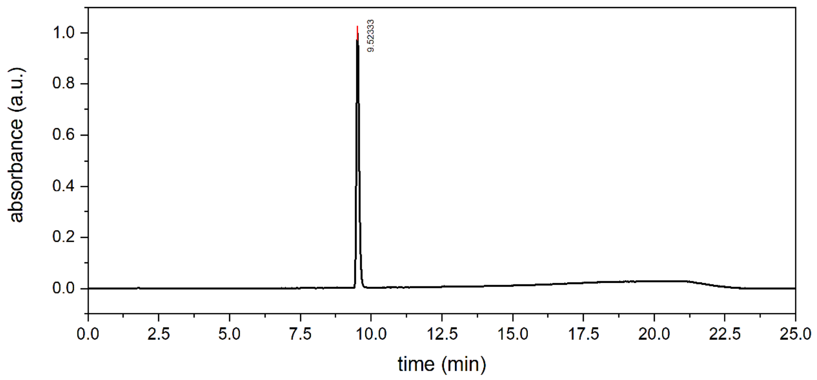

Figure S16: Analytical RP-HPLC chromatogram (System A) of compound **16**.

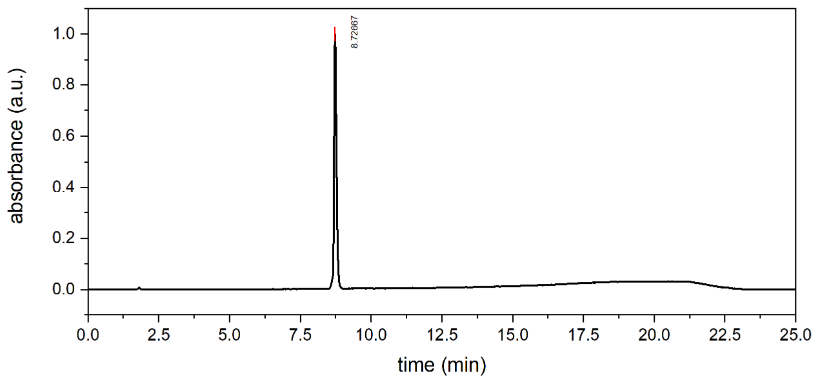

Figure S17: Analytical RP-HPLC chromatogram (System A) of compound **17**.

3. Mass Spectra of literature unknown compounds

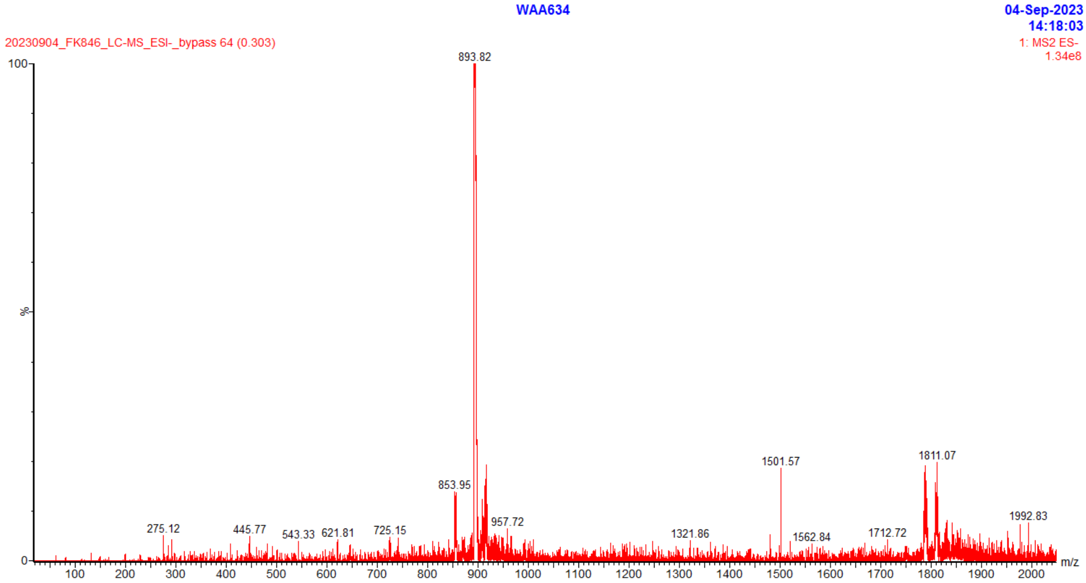

Figure S18: MS-Spectrum (ESI+) of compound **10**.

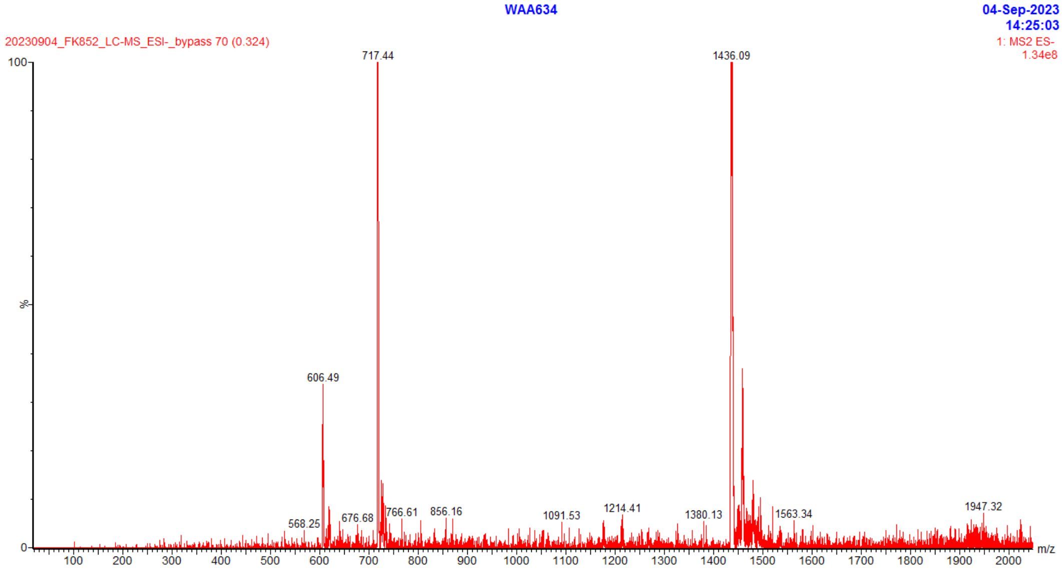

Figure S19: MS-Spectrum (ESI+) of compound **12**.

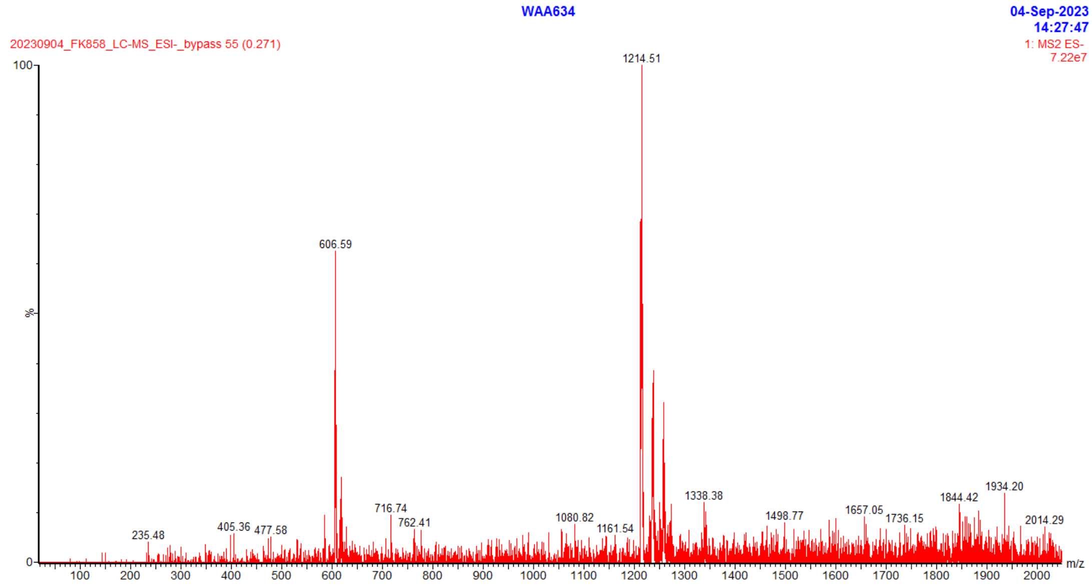

Figure S20: MS-Spectrum (ESI+) of compound **13**.

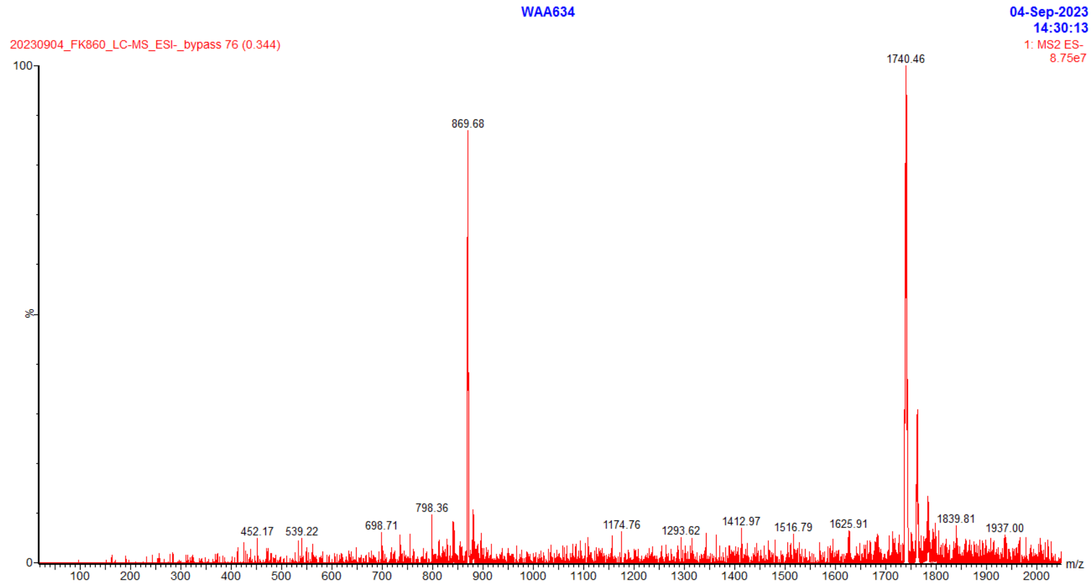

Figure S21: MS-Spectrum (ESI+) of compound **14a**.

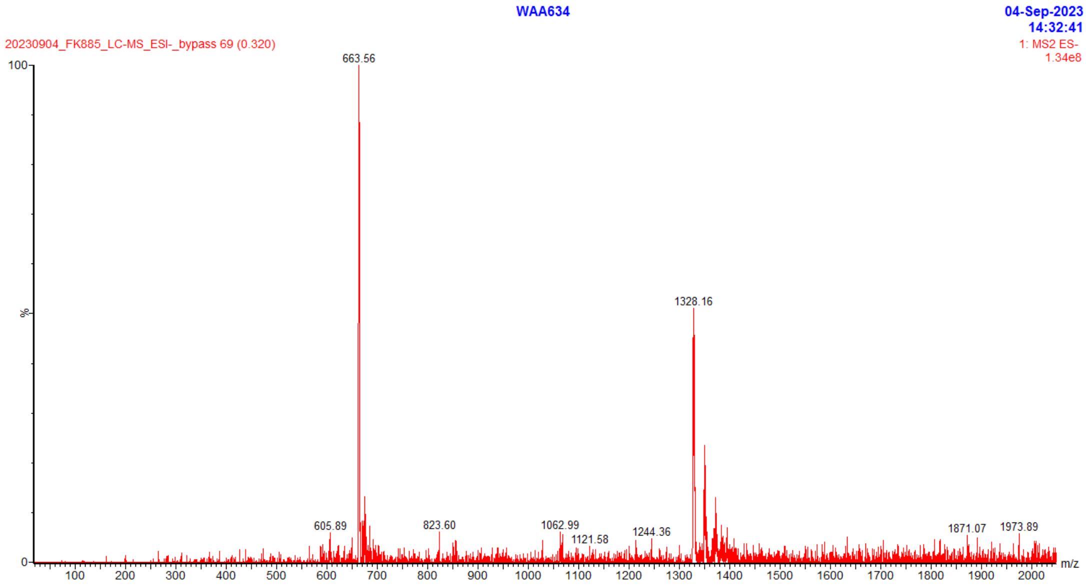

Figure S22: MS-Spectrum (ESI+) of compound **16a**.

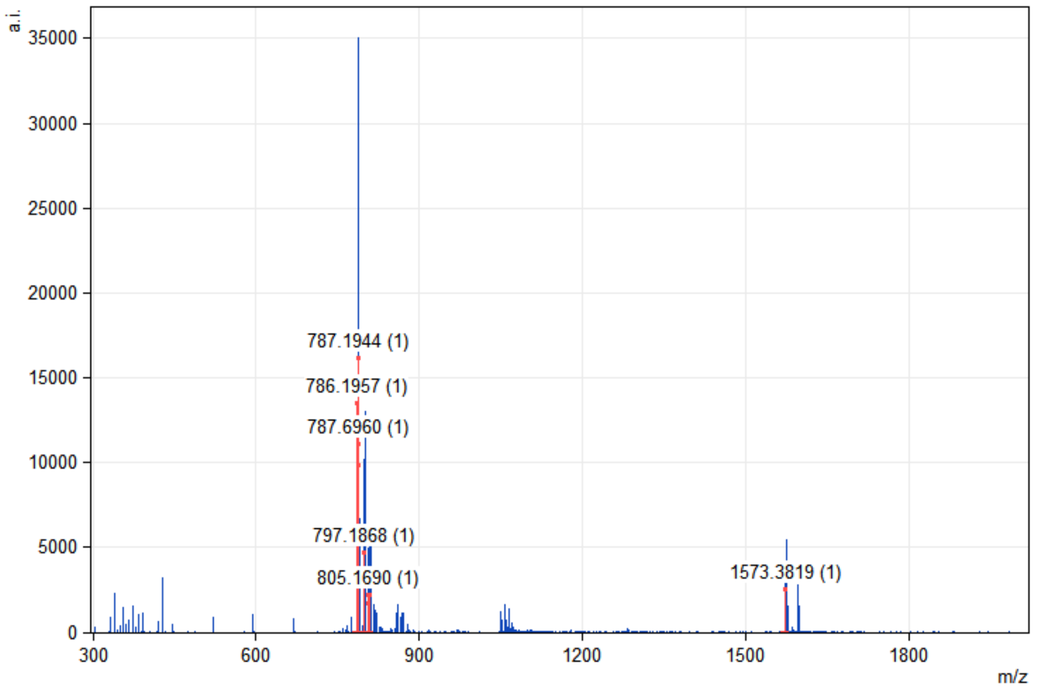

Figure S23: HR-MS-Spectrum (ESI+) of compound **14**.

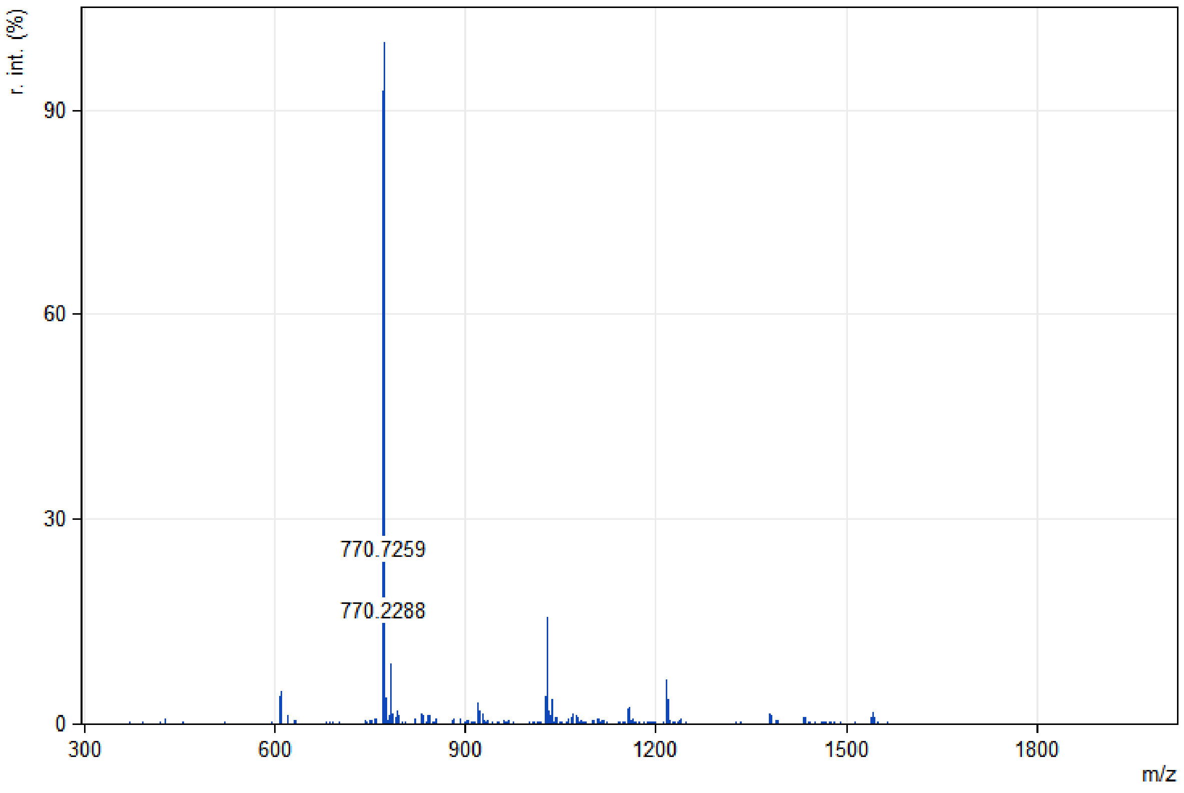

Figure S24: HR-MS-Spectrum (ESI+) of compound **15**.

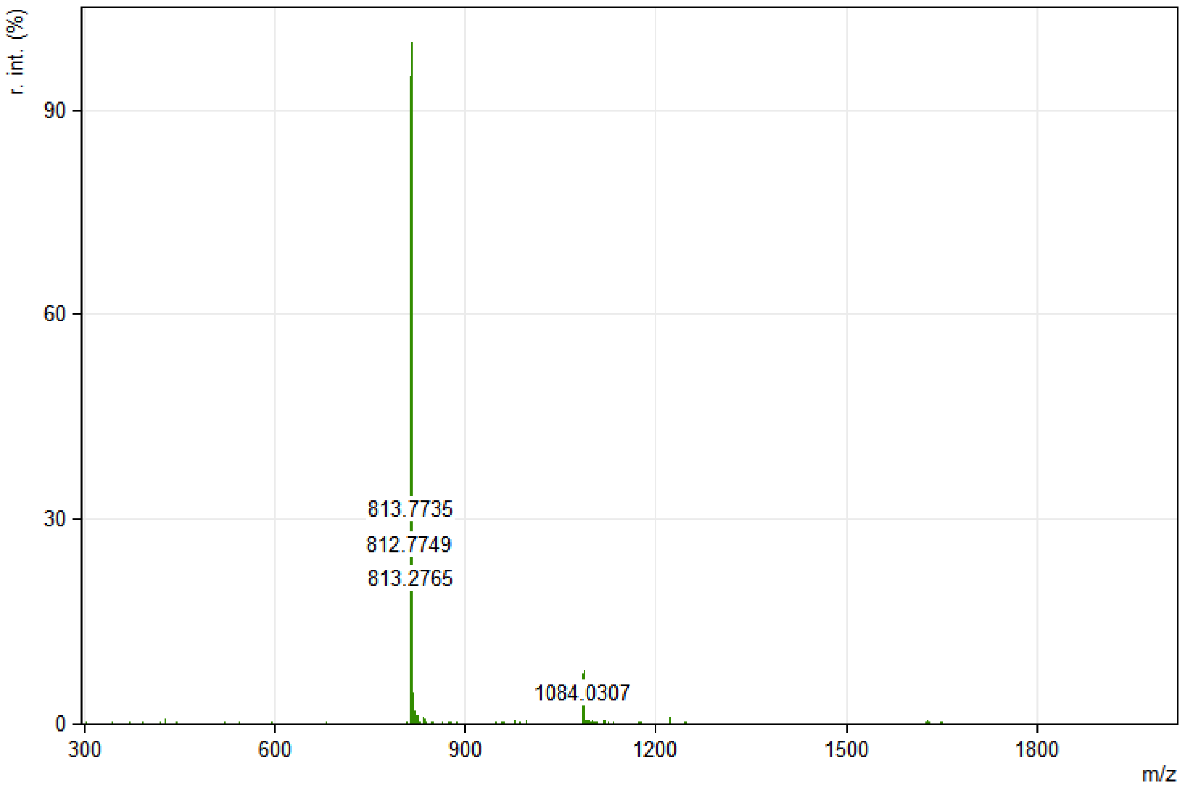

Figure S25: HR-MS-Spectrum (ESI+) of compound **16**.

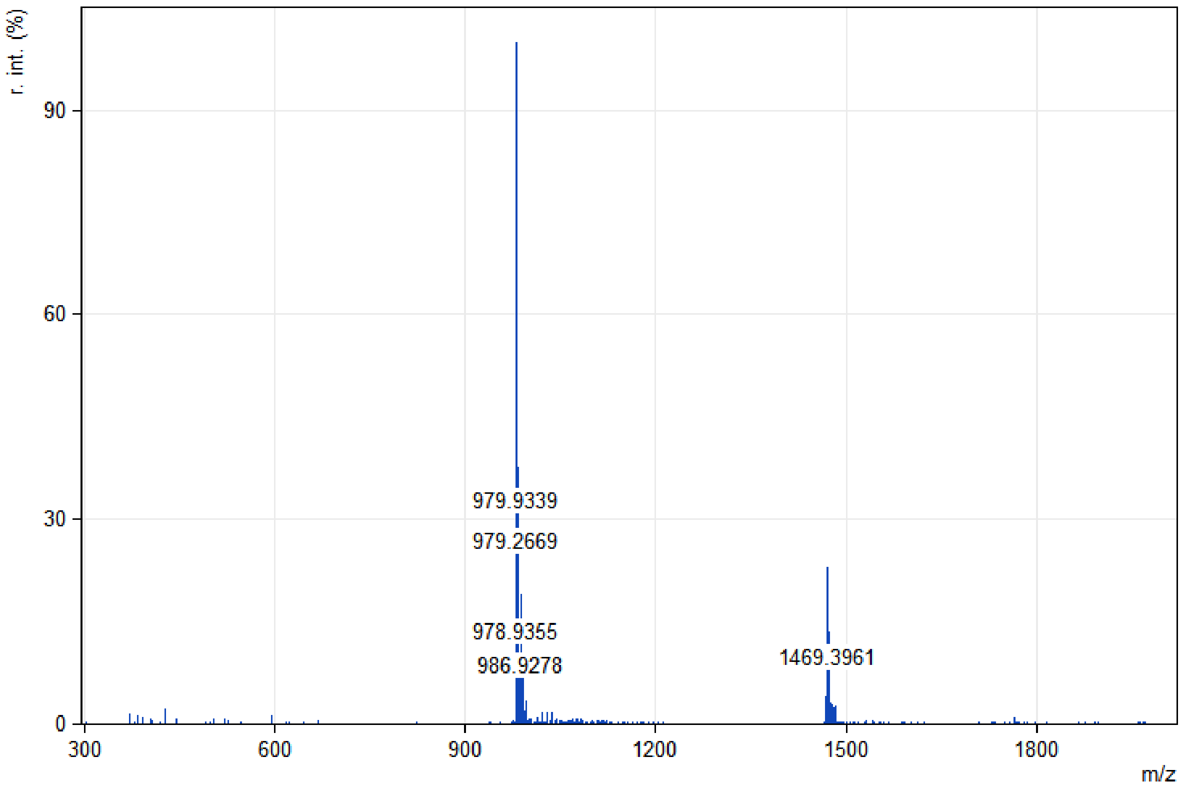

Figure S26: HR-MS-Spectrum (ESI+) of compound **17**.

4. Biological data

4.1 In vitro cell uptake

Both the target-positive (PC3 PD-L1) and negative cells (PC3 mock) were seeded at ~60,000 cells per well two days prior to the experiment. At the time of experiment, cells were then incubated with the respective radioligand at or close to the K_D (determined by saturation binding) in binding buffer. For each timepoint and radiotracer (30, 60, 120 and 240 min), a single plate was used (with triplicate measurements). Additional controls per plate included plastic retention, nonspecific binding [excess of unlabeled BMS-1166 (in DMSO, resulting in 0.03% v/v) for both PC3 PD-L1 and PC3 mock] and cells incubated with PBS only (protein control). Following incubation, cells were washed twice in ice-cold binding buffer, lysed in 500 µL of 0.1 M NaOH + 1% SDS and radioactivity was measured in a gamma counter (Perkin Elmer Wizard 1480) and decay corrected to a reference time (end of radiolabeling). Protein content was determined for each timepoint and cell type by BCA assay.

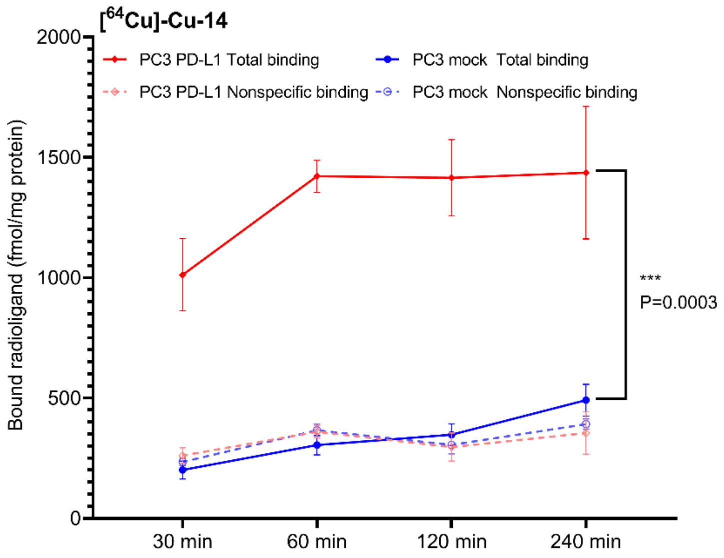

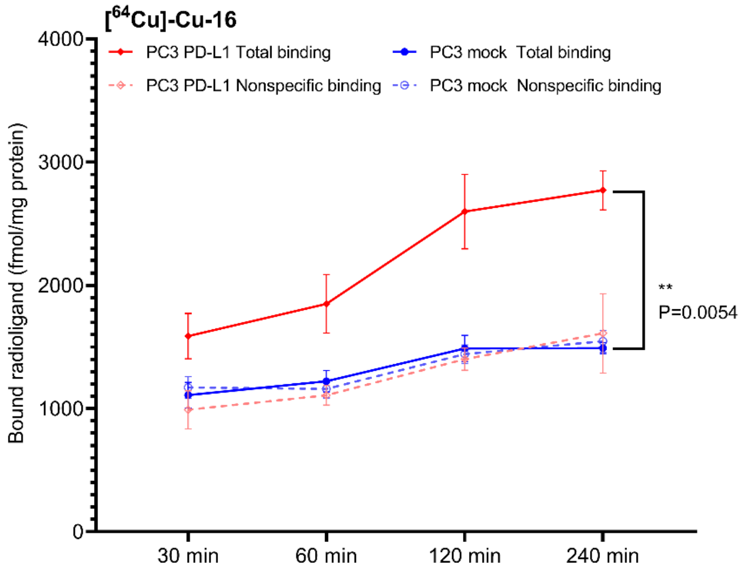

Figure S27: Cell uptake (fmol/mg protein) of [^{64}Cu]Cu-**14&16** into either PC3 PD-L1 or PC3 mock cells. Uptake of both [^{64}Cu]Cu-14&16 is significantly different (main effect of cell type) for PC3 PD-L1 vs. PC3 mock cells, as tested with a mixed-effects model. Binding to PC3 mock cells is similar to that of PC3 PD-L1 and PC3 mock cells blocked with an excess of unlabeled BMS-1166.

As expected from a small molecule in an *in vitro* setting, cellular uptake was relatively quick (Figure S27). [^{64}Cu]Cu-**14** uptake apparently reached a plateau by 60 min. In contrast, [^{64}Cu]Cu-**16** was approaching a plateau between 120 and 240 min. For [^{64}Cu]Cu-**16**, this could potentially involve nonspecific binding, as plastic retention was observed to be between 0.43% and 0.59% of tracer stock activity (increasing over time). [^{64}Cu]Cu-**14** was characterized by a 10 times lower plastic retention (0.03% of stock activity), which was stable over time.
When comparing target-positive (PC3-PD-L1) and target-negative cells (PC3 mock), a mixed-effects model (with Geisser-Greenhouse correction for sphericity) showed a significant main effect of time for [^{64}Cu]Cu-**14** [F (1.442, 5.286) = 11.47, P<0.0144] and [^{64}Cu]Cu-**16**: [F (1.239, 5.783) = 17.68, P<0.0051]). More importantly, there was a significant main effect of target expression, again for both compounds [^{64}Cu]Cu-**14** [F (1, 4) = 136.6, P<0.0003] and [^{64}Cu]Cu-**16** [F (1, 4) = 29.89, P<0.0054]). Uptake to PC3 mock cells was very similar to PC3 PD-L1 and PC3 mock cells preincubated with an excess of unlabeled BMS-1166. Taken together, these results further support the specificity of *in vitro* binding of both compounds to its target and the kinetics observed in vitro.

Table S1: *In vivo* standardized uptake values (SUV_{max} ± standard deviation) of ^{64}Cu-labeled compounds [^{64}Cu]Cu-**14**–**17** at 1.5 (1–2), 4.5 (4–5) and 24.5 (24–25) hours post injection (p.i.).

			**1-2 hour p.i.**		**4-5 hour p.i.**		**24-25 hour p.i.**	
			SUVmax (mean)	SD	SUVmax (mean)	SD	SUVmax (mean)	SD
**[^{64}Cu]Cu-14** n=2 each	tracer	PC3 PD-L1	1.71	0.04	1.26	0.37	1.52	0.51
		PC3 mock	0.86	0.16	0.50	0.12	1.49	0.25
		liver	3.41	0.19	1.54	0.19	1.41	0.02
		kidney	22.77	2.98	0.72	0.24	0.63	0.06
	tracer	PC3 PD-L1	1.48	0.44	0.78	0.10	1.53	0.19
	+500 nM	PC3 mock	1.19	0.35	0.48	0.13	1.05	0.11
	**14**	liver	1.25	0.13	0.98	0.16	0.99	0.23
		kidney	32.81	10.61	0.88	0.06	0.44	0.02
**[^{64}Cu]Cu-15** n=2 each	tracer	PC3 PD-L1	1.03	0.23	0.66	0.16	0.90	0.09
		PC3 mock	0.61	0.39	0.44	0.18	0.76	0.58
		liver	3.80	0.33	1.41	0.88	1.01	0.21
		kidney	3.26	0.98	0.68	0.10	0.48	0.08
	tracer	PC3 -L1	1.58	0.56	0.77	0.03	0.91	0.13
	+500 nM	PC3 mock	1.17	0.35	0.57	0.19	0.78	0.06
	**15**	liver	1.49	0.01	1.82	1.42	0.78	0.19
		kidney	7.41	4.18	0.78	0.04	0.51	0.06
**[^{64}Cu]Cu-16** n=3 tracer n=2 block	tracer	PC3 PD-L1	1.61	0.93	0.89	0.55	1.47	0.91
		PC3 mock	0.78	0.36	0.37	0.02	0.87	0.42
		liver	4.32	0.02	2.49	0.16	1.77	0.08
		kidney	16.18	15.52	1.37	0.30	0.90	0.18
	tracer	PC3 PD-L1	1.45	0.06	0.68	0.05	1.14	0.23
	+500 nM	PC3 mock	0.90	0.04	0.54	0.11	1.01	0.16
	**16**	liver	1.13	0.04	0.84	0.15	0.85	0.29
		kidney	7.93	5.69	1.28	0.22	1.02	0.45
**[^{64}Cu]Cu-17** n=4	tracer	PC3 PD-L1	0.49	0.11	0.81	0.27	2.47	1.04
		PC3 mock	0.31	0.06	0.50	0.15	1.18	0.34
		liver	17.03	1.48	14.35	1.26	8.62	0.84
		kidney	1.32	0.47	2.18	0.80	2.40	0.60
